# Adiponectin-mimetic novel nonapeptide rescues aberrant neuronal metabolic-associated memory deficits in Alzheimer’s disease

**DOI:** 10.1186/s13024-021-00445-4

**Published:** 2021-04-13

**Authors:** Tahir Ali, Shafiq Ur Rehman, Amjad Khan, Haroon Badshah, Noman Bin Abid, Min Woo Kim, Myeung Hoon Jo, Seung Soo Chung, Hyoung-gon Lee, Bart P. F. Rutten, Myeong Ok Kim

**Affiliations:** 1 Division of Applied Life Science (BK 21 Four), College of Natural Science, Gyeongsang National University, Jinju, 52828 Republic of Korea; 2 Department of Physiology, College of Medicine, Yonsei University, Seoul, 120-752 Republic of Korea; 3 Department of Biology, The University of Texas at San Antonio, San Antonio, USA; 4 Translational Neuroscience and Psychiatry, School for Mental Health and Neuroscience (MHeNs), Maastricht University Medical Centre, Maastricht, Netherlands

**Keywords:** Alzheimer’s disease (AD), Brain metabolic disorders, Neuronal adiponectin receptor 1 (AdipoR1), Adiponectin-mimetic novel nonapeptide (Os-pep), AdipoR1/AMPK signaling, Neuronal insulin resistance, Insulin signaling, Synaptic and memory deficits

## Abstract

**Background:**

Recently, we and other researchers reported that brain metabolic disorders are implicated in Alzheimer’s disease (AD), a progressive, devastating and incurable neurodegenerative disease. Hence, novel therapeutic approaches are urgently needed to explore potential and novel therapeutic targets/agents for the treatment of AD. The neuronal adiponectin receptor 1 (AdipoR1) is an emerging potential target for intervention in metabolic-associated AD. We aimed to validate this hypothesis and explore in-depth the therapeutic effects of an osmotin-derived adiponectin-mimetic novel nonapeptide (Os-pep) on metabolic-associated AD.

**Methods:**

We used an Os-pep dosage regimen (5 μg/g, i.p., on alternating days for 45 days) for APP/PS1 in amyloid β oligomer-injected, transgenic adiponectin knockout (Adipo−/−) and AdipoR1 knockdown mice. After behavioral studies, brain tissues were subjected to biochemical and immunohistochemical analyses. In separate cohorts of mice, electrophysiolocal and Golgi staining experiments were performed. To validate the *in vivo* studies, we used human APP Swedish (swe)/Indiana (ind)-overexpressing neuroblastoma SH-SY5Y cells, which were subjected to knockdown of AdipoR1 and APMK with siRNAs, treated with Os-pep and other conditions as per the mechanistic approach, and we proceeded to perform further biochemical analyses.

**Results:**

Our *in vitro* and *in vivo* results show that Os-pep has good safety and neuroprotection profiles and crosses the blood-brain barrier. We found reduced levels of neuronal AdipoR1 in human AD brain tissue. Os-pep stimulates AdipoR1 and its downstream target, AMP-activated protein kinase (AMPK) signaling, in AD and Adipo−/− mice. Mechanistically, in all of the *in vivo* and *in vitro* studies, Os-pep rescued aberrant neuronal metabolism by reducing neuronal insulin resistance and activated downstream insulin signaling through regulation of AdipoR1/AMPK signaling to consequently improve the memory functions of the AD and Adipo−/− mice, which was associated with improved synaptic function and long-term potentiation via an AdipoR1-dependent mechanism.

**Conclusion:**

Our findings show that Os-pep activates AdipoR1/AMPK signaling and regulates neuronal insulin resistance and insulin signaling, which subsequently rescues memory deficits in AD and adiponectin-deficient models. Taken together, the results indicate that Os-pep, as an adiponectin-mimetic novel nonapeptide, is a valuable and promising potential therapeutic candidate to treat aberrant brain metabolism associated with AD and other neurodegenerative diseases.

**Supplementary Information:**

The online version contains supplementary material available at 10.1186/s13024-021-00445-4.

## Background

Neurodegenerative disorders have become one of the greatest challenges and threats to our society because the incidence of these diseases is high in the middle-age and elderly populations. Despite the huge advancement in the health sciences, no therapeutics are available for most neurodegenerative diseases [1, 2]. Currently, the most important and debilitating neurodegenerative diseases are Alzheimer’s disease (AD), Parkinson’s disease (PD), and amyotrophic lateral sclerosis (ALS). In all these neurodegenerative diseases, AD is a prime focus for researchers and clinicians, as recent statistical data on AD reported that globally, more than 50 million individuals are affected with AD, and this number will triple by 2050. The worldwide cost of AD is approximately one trillion dollars annually, which will reach two trillion by the year 2030 [3]. Therefore, it is of the utmost importance to reveal the underlying mechanisms and explore potential novel therapeutic targets/agents for the treatment of AD. Recently, researchers have shown great interest in brain metabolic disorders in neurodegenerative diseases, and studies have suggested that aberrant brain metabolism plays a role in the disease-modifying effects of effective therapeutic substances against neurodegenerative diseases such as AD [4, 5]. Several established studies have reported that impaired insulin activities and neuronal insulin resistance are associated with AD pathologies, while regulation of these factors prevents AD [6–10]. Among brain metabolic perturbations that regulate neuronal insulin resistance and insulin signaling, the signaling of adiponectin and its receptor are among the most recent and indispensable research areas. These signaling pathways have been altered in the brain and implicated in metabolic-associated neurological disorders, and they may be a contributing risk factor for AD and other neurodegenerative diseases. Subjects with adiponectin deficiency developed insulin resistance in the brain and impaired downstream insulin signaling, leading to AD pathology*.* Adiponectin has been reported to function as an anti-diabetic, insulin-sensitizing, anti-inflammatory and antioxidant agent and to provide protection against metabolic-associated AD [11–16]. Waragai et al reported the reduction of adiponectin in AD patients and suggested that reduced adiponectin signaling is involved in AD [17]. Importantly, adiponectin signaling through AdipoR1 has beneficial effects on brain metabolism via AMP-activated protein kinase (AMPK) activation and is consequently implicated in the regulation of energy balance and metabolism as well as multiple other functions, including sensitization of the insulin receptor signaling pathway, stimulation of mitochondria biogenesis, and suppression of inflammation [18–22]. Hence, adiponectin is an interesting endogenous therapeutic candidate for the prevention of metabolic-associated neurodegenerative diseases such as AD [14–16, 23], and future studies should investigate mimetics of adiponectin that will also act as adiponectin receptor agonists because the conversion of full-length adiponectin into an effective drug is limited due to its size. However, there have been several recent reports that the adiponectin paradox associated with other chronic diseases might be involved in the pathogenesis of AD. In other experimental approaches and studies, adiponectin has been shown to be detrimental in conditions such as chronic heart and chronic kidney diseases, which gives rise to the adiponectin paradox [24–28]. Recently, epidemiological and research studies have shown that high serum levels of adiponectin are associated with low skeletal muscle mass, low muscle density, and poor physical functioning, which are ultimately implicated in disability and mortality [29–31]. In contrast, in the brain, adiponectin is protective against central nervous system (CNS)-neurotoxic agents and conditions such as 1-methyl-4-phenyl-pyridinium (MPP+), kainic acid acid-induced excitotoxicity, *in vitro* amyloid beta (Aβ)-induced toxicity and synuclein-induced neurodegeneration [22, 32–35]. Adiponectin reduces Aβ oligomer (AβO)-mediated neuroinflammation [36]. Additionally, our group discovered the biological and therapeutic effects of osmotin, a plant protein and homologue of adiponectin [37, 38]. Osmotin was protective in Aβ and transgenic AD mouse models [37–40]. We also confirmed that osmotin acts as an anti-inflammatory, anti-diabetic and anti-obesity agent [41, 42]. Therefore, adiponectin-based mimetic therapeutic approaches should be closely investigated for all relevant conditions and experimental systems. Of note, we aimed to test the therapeutic effect of an adiponectin-mimetic nonapeptide (C-T-Q-G-P-C-G-P-T (Os-pep)) in early-stage AD mouse models as well as in adiponectin-deficient mice, which is a model that was recently found to show AD pathology.

Several ligands for adiponectin receptors have been reported, including osmotin, which reduces AD pathology [16, 37–41]. However, osmotin has faced the same problems and limitations as adiponectin; due to its large size, osmotin has been limited in its efficacy in translational studies. Therefore, as a translational strategy, we extended the research approach by utilizing the osmotin-based small adiponectin-mimetic Os-pep, which is a very structurally stable peptide that interacts with the adiponectin recognition site on AdipoR1 and shows similar activity as adiponectin and osmotin [37, 43–45]. Considering the evidence that Os-pep mimics adiponectin, Os-pep may also have significant potential therapeutic value for the prevention of metabolic-associated AD. It has been recently reported that aberrant adiponectin and AdipoR1/AMPK signaling are associated with impaired insulin signaling and brain insulin resistance in AD-associated pathology [14–16]. Furthermore, recent studies have highlighted the mechanistic and potential therapeutic effects of adiponectin and adiporon, which are stimulators of adiponectin receptors, on AD through the activation of metabolic cascades such as the AdipoR1/AMPK and downstream insulin signaling pathways [14–17, 22]. Herein, we hypothesize that novel Os-pep acts as an adiponectin-mimetic peptide to activate AdipoR1/AMPK signaling and subsequently rescue aberrant neuronal metabolism by reducing neuronal insulin resistance and activating downstream insulin signaling, which leads to improvements in synaptic and memory functions in AD and adiponectin knockout (Adipo^−/−^) mice.

## Materials & methods

We purchased the custom-synthesized 9-amino acid Os-pep from Peptron (Yuseong-daero, Daejeon, South Korea), which synthesized and characterized the Os-pep according to previously published protocols [44]. The purity (98%) of the peptide was analyzed and characterized using reverse-phase HPLC. Further, some materials and methods are described in the supplementary material.

### Mouse genotyping and grouping and optimization of the dosage regimen of Os-pep

To optimize and analyze the toxicity and effective dose of Os-pep, we performed a separate group of studies using wild-type (WT) male C57BL/6 N mice to examine the toxicity of Os-pep *in vivo*. The WT mice were rearranged into 5 groups (*n* = 5 mice/group). Os-pep was administered in bolus dosages of 10, 20, 30, 40 and 50 mg/kg in normal saline to the respective groups, and the Veh-injected mice received the same amount of normal saline as WT mice. We checked for signs of systemic toxicity, such as head tilt, tremor, abnormal physical activity and squinting, for 1 week in WT mice that had been treated with i.p. bolus doses of Os-pep (10, 20, 30, 40 and 50 mg/kg).

Male transgenic adiponectin-deficient (Adipo^−/−^) and double transgenic (APPswe, PSENdE9) 85Dbo/Mmjax amyloid precursor protein and presenilin 1 (APP/PS1) AD mice were purchased from the Jackson Laboratory (Bar Harbor, ME). WT C57BL/6 N male mice of the same age were purchased from Samtako Bio (South Korea). The mice were kept in the university animal housing facility under a 12-h (h)/12-h light/dark cycle at 23 °C in 60 ± 10% humidity with free access to water and food. Ten-month-old AβO-injected and APP/PS1 mice and 18-month-old Adipo^−/−^ mice were carried to the specified injection and behavioral room and allowed to acclimate for a few days. AβO or vehicle (Veh) (normal saline) was injected as previously described [38]. Seven days after the intracerebroventricular (i.c.v.) AβO and Veh injections, the mice were divided into the respective groups: mice that received normal saline (Veh only) by an intraperitoneal (i.p.) injection (control), mice that received normal saline (Veh only) by the i.c.v. route (sham), mice that received AβO by the i.c.v. route (AβO), mice that received AβO by the i.c.v. route that were also treated with Os-pep (AβO + Os-pep) and mice treated with Os-pep alone (Os-pep).

The remaining transgenic and WT mice were grouped as follows: (1) Veh-injected WT, (2) Veh-injected APP/PS1, and (3) Os-pep-treated APP/PS1 mice. The Adipo^−/−^ mice were grouped as follows: (1) Veh-injected WT, (2) Veh-injected Adipo^−/−^, and (3) Os-pep-treated Adipo^−/−^ mice. The grouping and treatment of high-fat diet (HFD)-fed animals and the animals used for pharmacokinetic studies are described in the supplementary material.

Os-pep was dissolved in double-deionized distilled water and then prepared in normal saline to obtain the final administered concentration. The AβO, APP/PS1, Adipo^−/−^ and WT mice were injected i.p. with Os-pep at a dosage of 5 μg/g on alternating days for 45 days. WT, Veh-injected transgenic-APP/PS1 and Adipo^−/−^ mice were injected with the same volume of double-deionized water and normal saline. The WT control and sham mice as well as the Veh-injected AβO, APP/PS1, Adipo^−/−^ and HFD mice received the same volume of double-deionized distilled water or normal saline.

### Generation of AdipoR1 knock down mice

In a separate cohort of study, we used the polyethylenimine (PEI)-based scramble shRNA and functional shRNA mediating knockdown of AdipoR1 genes in the AβO-injected mice as previously described [21].

### Behavioral studies

To evaluate the learning and memory functions of the mice, we performed the Morris water maze (MWM) and Y-maze tests. After completion of the Os-pep dosage regimen, behavioral studies were performed on AβO-injected, AβO-injected and scramble and functional AdipoR1 shRNA-treated, APP/PS1 and Adipo^−/−^ mice (*n* = 13/group) using the MWM and Y-maze tests. Notably, the separate groups of APP/PS1 and Adipo^−/−^ mice (*n* = 5–6/group) used for the LTP assay were also subjected to behavioral analyses. The mice were randomly selected, and the operators were unaware of the genotype and treatment details for the behavioral study. The MWM and Y-maze tests were performed as previously described [38, 39]*.*

### Electrophysiology experiments

A separate group of animals were used for the *in vitro* electrophysiology analysis. The APP/PS1 and Adipo^−/−^ mice received i.p. injections of 5 μg/g Os-pep on alternating days for 45 days. The WT and Veh-injected APP/PS1 and Adipo^−/−^ mice received the same volume of bi-deionized distilled water and normal saline, respectively (n = 5–6/group). After the dosage regimen was administered, the mice were subjected to behavioral tests. Then, the mice were anesthetized with isoflurane, and the brains were rapidly cooled via transcardial perfusion with ice-cold sucrose artificial cerebrospinal fluid (CSF). The brains were removed and placed in ice-cold sucrose artificial CSF. Coronal slices were prepared and incubated in artificial CSF at 35 °C for 30 min for recovery. The slices were then incubated with artificial CSF at room temperature (23 °C–25 °C) for 1–4 h before being placed in the recording chamber for the experiments. The standard artificial CSF contained (mM): 119 NaCl, 2.5 KCl, 2.5 CaCl_2_, 1.3 MgSO_4_, 1.0 NaH_2_PO_4_, 26.2 NaH_2_CO_3_, 11.0 glucose, 2.0 Na pyruvate, and 1.0 Na ascorbate saturated with 95% O_2_/5% CO_2_. The sucrose artificial CSF contained (mM): 198 sucrose, 2.5 KCl, 1 NaH_2_PO_4_, 26.2 NaHCO_3_, 11 glucose, 2.0 Na pyruvate, and 1.0 Na ascorbate saturated with 95% O_2_/5% CO_2_. All experiments were conducted at 27 °C–29 °C. For electrophysiological experiments, pipette electrodes with 3–6 MΩ resistance were used, and whole-cell recordings were obtained from neurons using infrared (IR)-differential interference contrast (DIC) optical guidance. The CA3 and DG regions were excised immediately before the LTP experiments to isolate the CA1 lesion. Stimuli were applied to the Schaffer-collateral (SC) pathway using a concentric bipolar electrode located 100–200 mM from the soma of the recorded cell. The whole-cell recording solution comprised (mM): 135 Cs methanesulfonate, 8 NaCl, 10 HEPES, 0.5 EGTA, 4 Mg-ATP, 0.3 Na-GTP and 5 QX-315 Cl (pH 7.25 with CsOH, 285 mOsm). Cells were maintained at − 70 mV during recordings unless indicated otherwise. Recordings were collected using an Axopatch ID (Molecular Devices, Sunnyvale, CA) and were digitized at 10 KHz and filtered at 2 KHz. The input resistance and series resistance were monitored continuously during the recordings. The test stimulation in all EPSC experiments was set to 0.1 Hz and was 0.2 ms in duration, and its intensity (100–900 μA) was adjusted to induce EPSCs with amplitudes ranging from 50 to 100 pA at a holding potential of − 70 mV. In the LTP experiments, the baseline EPSCs were measured for 3 min prior to the introduction of the paired stimuli (2 Hz, 2 min stimulus and postsynaptic depolarization to 0 mV). After the introduction of the paired stimuli (2 Hz, 2 min stimulus and postsynaptic depolarization to 0 mV), EPSCs were measured every 10 s for 30 min.

Similarly, in another separate group of studies, normal mice were used for the *in vitro* electrophysiology analysis. The AβO-injected, AβO-injected and scramble shRNA-treated, and AβO-injected mice with shRNA-mediated silencing of the AdipoR1 gene received i.p. injections of 5 μg/g Os-pep on alternating days for 45 days. The control (Veh-injected) and AβO-injected mice received the same volume of bi-deionized distilled water and normal saline, respectively (*n* = 5–6/group). After the dosage regimen was administered, the mice were subjected to behavioral tests. Furthermore, to investigate the CA1 circuit via the Schaffer collateral (SC) input in the hippocampus, hippocampal brain slices (400 mM-thick) were prepared from adult mice. Briefly, after the mice were anesthetized with isoflurane, the brain was rapidly cooled via transcardiac perfusion with ice-cold sucrose artificial cerebrospinal fluid (aCSF). The brain was removed and placed in ice-cold sucrose aCSF. Coronal slices were prepared and were incubated in aCSF at 35 °C for 30 min to allow recovery. Slices were then incubated in artificial CSF at room temperature (23 °C–25 °C) for 1–4 h before being placed in the recording chamber for experiments. The standard aCSF contained (mM): 124 NaCl, 2.5 KCl, 2.5 CaCl_2_, 1.3 MgSO_4_, 1.0 NaH_2_PO_4_, 26.2 NaH_2_CO_3_, 11 glucose, 2 Na pyruvate, and 1 Na ascorbate saturated with 95% O_2_/5% CO_2_. The sucrose aCSF contained (mM): 195.5 sucrose, 2.5 KCl, 2.5 CaCl_2_, 1.3 MgSO_4_, 1 NaH_2_PO_4_, 26.2 NaHCO_3_, 11 glucose, 2 Na pyruvate, and 1 Na ascorbate saturated with 95% O_2_/5% CO_2_. All experiments were conducted at 27 °C – 29 °C. For the electrophysiological experiments, pipette electrodes with 3–6 MΩ resistance were used, and whole-cell recordings were obtained from neurons under visual guidance using infrared (IR)-differential interference contrast (DIC) optics. The CA3 and DG regions were removed immediately prior to starting the LTP (long-term potentiation) experiments to isolate the CA1 lesions. Stimuli were applied to the Schaffer collateral (SC) pathway. Field excitatory postsynaptic potentials (fEPSPs) were recorded in the CA1 stratum radiatum by using an extracellular glass pipette (3–5 MΩ) filled with aCSF. Schaffer collateral/commissural fibers in the stratum radiatum were stimulated using a concentric bipolar electrode placed 200–300 μm away from the recording pipette. The test stimulation in all fEPSP experiments was performed at 0.1 Hz and was 0.2 ms in duration. For long-term potentiation (LTP), a voltage that elicited 50% of the maximum fEPSP was used. Baseline synaptic responses were recorded for 20 min, and then LTP was induced by two high-frequency stimulation trains (100 Hz, 1 s duration) separated by a 20 s intertrain interval. Recordings were made every 30 s for 2 h using an Axopatch 1D amplifier (Molecular Devices, Sunnyvale, CA) and were digitized at 10 KHz and filtered at 2 KHz with Digidata 1322A and pClamp 9.0 software (Molecular Devices, Sunnyvale, CA).

### Human APPswe/ind plasmid

The human APPswe/ind gene in the pCAX mammalian expression vector with transcriptional control under the SV-40 promoter/enhancer was obtained from Addgene; this sequence was deposited in the APP repository by Dennis Solkoe (Plasmid 30,145). The plasmid was amplified in the bacterial strain DH5alpha in Luria-Bertani media with 50 μg/ml ampicillin. The plasmid was purified with the Qiagen Plasmid Midi Kit (Cat # 12143) and confirmed by restriction digestion with the EcoRV restriction enzyme.

### Human neuroblastoma SH-SY5Y cells cultured with overexpression of the human APPswe/ind plasmid, siRNA plasmid and drug treatment

Human neuroblastoma SH-SY5Y cells were seeded (in 6-well culture plates for immunoblotting and in chamber slides for immunofluorescence) in DMEM (Dulbecco’s modified Eagle medium) containing 10% fetal bovine serum (FBS) and 1% antibiotics (penicillin-streptomycin) and incubated at 37 °C in humidified air containing 5% CO_2_. Once the cells reached 70–80% confluence, the cells were transfected using the pCAX vector containing the APPswe/ind gene using Lipofectamine 3000 (Life Technologies) according to the provider’s protocols. AdipoR1 siRNA (h) (SC-60123) and AMPK siRNA (h) (SC-45312) (Santa Cruz Biotechnology, Inc.) were used at a concentration of 10 μM per transfection and expressed for 72 h according to the manufacturer’s protocol (Santa Cruz Biotechnology, Inc.). A negative siRNA (Ambion, Thermo Fisher Scientific) was used as a control. Seventy-two hours after the transfections, the cells were exposed to Os-pep (10 μM) for 12 h and then subjected to immunoblotting and immunofluorescence analyses.

However, in the case of drug treatment, SH-SY5Y cells (2 × 10^4^/ml) were cultured in 35-mm dishes in DMEM containing FBS (10%) and antibiotics (1%) at 37 °C in humidified air containing 5% CO2. After reaching 70% confluence, the SH-SY5Y cells were treated with AβO (1 μM), AβO (1 μM) + Os-pep (10 μM), Os-pep (10 μM), or DMSO (0.01%) as a control for 12 h and subjected to immunoblot and immunofluorescence analyses.

### Immunoblotting assaypyramidal neuronal

The protein concentrations of the homogenates of mouse brain tissue and cell lysates *in vitro* were measured using a Bio-Rad protein assay kit (Bio-Rad Laboratories, Hercules, CA), and immunoblotting was performed as we previously described [38], using primary antibodies (detailed information described in the supplementary Table 1). Western blotting membranes have been cut prior to original western blot images and original western blot images are available at supplementary material.

### Confocal microscopy and stereological analyses

Human postmortem paraffin-embedded human brain tissue from healthy aged patients and AD aged patients of the same age were obtained from the Department of Pathology, Case Western Reserve University School of Medicine, USA. Human brain tissue samples (10 μm-thick sections) on gelatin-coated slides were deparaffinized three times with absolute xylene (5 min for each wash) and rehydrated with graded ethyl alcohol (100 to 70%). After deparaffinization of the human brain tissue sections, samples were prepared from the mouse brain as we previously described [38], and chamber slides containing cells were washed twice with phosphate-buffered saline (PBS) (0.01 M) for 10 min. After washing, the slides containing samples were incubated for 1 h with 2% normal serum as a blocking solution and 0.3% Triton X-100 in PBS. After blocking, the slides were incubated with primary antibodies (rabbit-p-IRS-1 (Ser 636) and mouse Aβ (B4) from Santa Cruz Biotechnology, Dallas, TX, USA) and rabbit AdipoR1 (Abcam) diluted 1:100 in blocking solution overnight at 4 °C. After primary antibody incubation, the sections were washed twice for 5 min each and incubated for 2 h with tetramethylrhodamine isothiocyanate (TRITC)/fluorescein isothiocyanate (FITC)-conjugated secondary antibody (1:50) from Santa Cruz Biotechnology, Inc. Furthermore, following incubation with the TRITC/FITC-conjugated antibody (1:50), the slides were incubated overnight with the second required primary antibody. Following incubation with the second primary antibody, the sections were incubated with another secondary antibody (FITC/TRITC-conjugated (1:50)) for 2 h at room temperature. Coverslips were mounted with DAPI along with Dako fluorescent mounting medium (Molecular Probe, Eugene, OR). The double immunofluorescence and single immunofluorescence confocal images were captured through a laser confocal microscope (FV10-ASW 3.1 Viewer, Olympus, Tokyo, Japan). Five images per section (tissue) were captured (by an operator blinded to the experimental groups) from every respective group. Next, the real confocal images were converted to tagged image file format (TIF). The quantification of the immunofluorescence intensity in the same region of the cortex/total area and hippocampus/total area in the TIF images for all groups was performed using ImageJ software with the method described below. The TIF image background was optimized according to the threshold intensity, and the immunofluorescence intensity was analyzed at the specified threshold intensity for all groups and was expressed as the integrated density of the samples relative to that of the control.

### Golgi staining and morphological analyses of hippocampal pyramidal neuronal cells

In a separate cohort of studies, we performed Golgi staining and morphological analysis of hippocampal pyramidal neuronal cells and were investigated as accordingly our previous protocol [40].

### Data and statistical analyses

Histograms and graphs were generated using GraphPad Prism 5 (GraphPad Software, San Diego, CA). The cumulative probability curves of mEPSCs were calculated using the Clampfit 9.0 software and GraphPad Prism 4. Statistical analyses were performed using one-way/two-way analysis of variance (ANOVA) followed by two-tailed independent Student’s *t*-test, Bonferroni’s multiple comparisons test or Tukey’s post hoc test, as applicable, for comparisons among the groups. The data are presented as the means ± SEM of the three independent experiments. The escape latencies in the behavioral tests were analyzed using a two-way ANOVA, with training days as the repeated measurements. The operators and investigators who performed the behavioral, biochemical, immunohistochemical and the electrophysiological assays and the quantitative analyses thereof were blinded to the dosage regimen and grouping of animals, as well as to the *in vitro* experimental drug treatments and grouping. The number of animals/human tissue per experiment and the number of experiments per group in the *in vivo* and *in vitro* experiments are presented in each section and panel of the figure legend. Statistical significance = *P < 0.05*. * Significantly different compared with the WT Veh-injected group; **#** significantly different compared with the AβO-injected, Veh-injected APP/PS1, Adipo^−/−^ and HFD mice. For all *in vitro* results, comparisons among groups are indicated by bars.

## Results

### Os-pep activated AdipoR1/AMPK signaling both in *in vitro* and *in vivo* AD models and Adipo^−/−^ mice

**Fig. 1:**
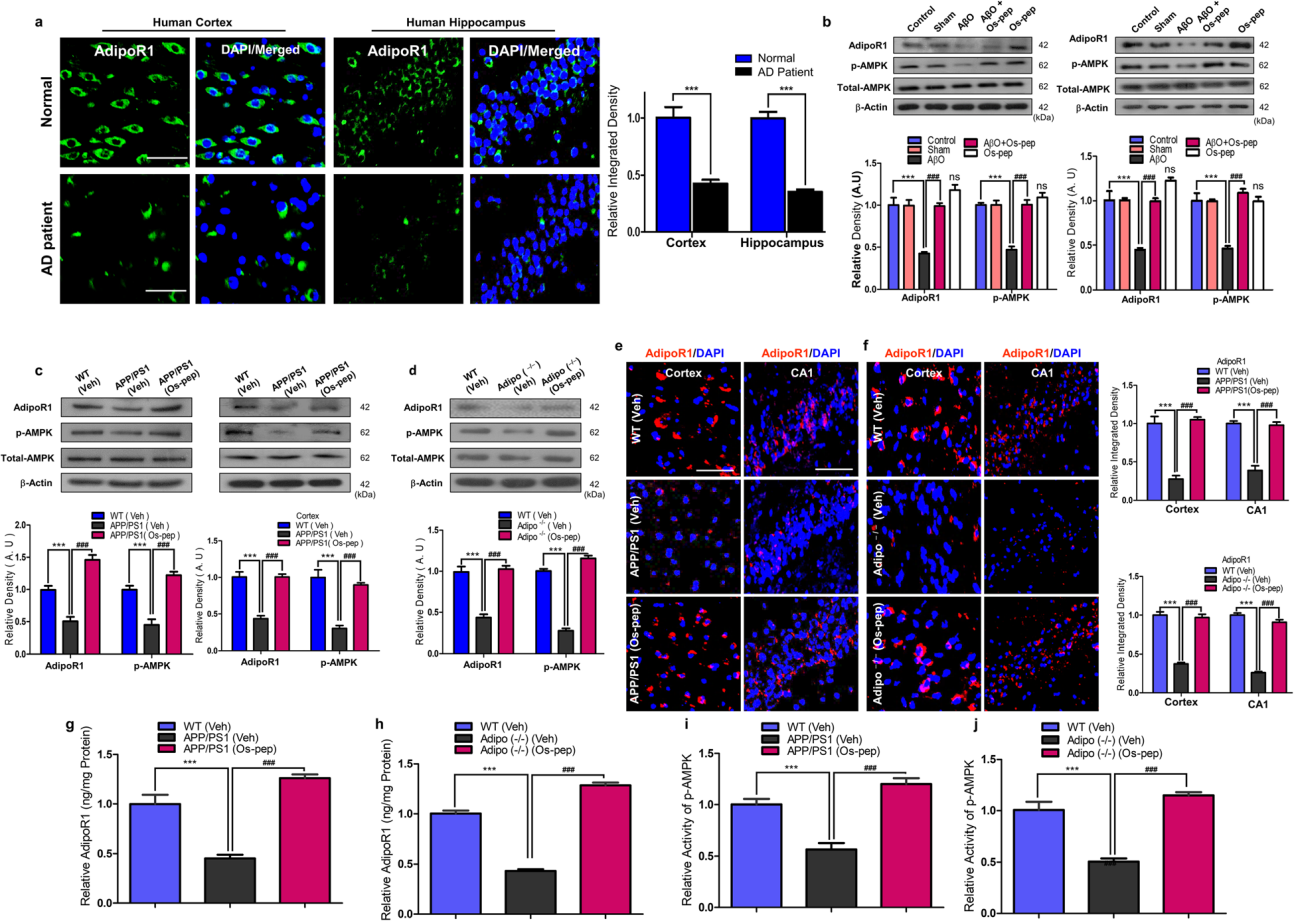
Os-pep stimulated AdipoR1/p-AMPK signaling in in vivo AD models and Adipo^−/−^ mice **(a)** The AdipoR1 immunofluorescence reactivity in the normal aged and AD aged patients’ brain tissue (AdipoR1; Green, DAPI, Blue). Magnified 40X. Scale bar = 50 μm. The data are indicated as the ± SEM for *n* = 5 human brain per group and the number of independent experiments = 3. Significance = ***p < 0.001; student’s t test **(b-d)** Immunoblotting and quantification of AdipoR1 and p-AMPK/total-AMPK expression in the brain tissue of AβO, APP/PS1 and Adipo^−/−^ mice. The data are expressed as the means ± SEM for the indicated proteins in vivo (*n* = 8 mice/group) and the number of independent experiments = 3. Significance = ***p < 0.001; ###*p* < 0.001; One-way ANOVA followed by Turkey’s post hoc test. ns, indicated that there is no significant difference among the control, sham and Os-pep treated groups (**e, f)** Immunofluorescence staining for AdipoR1 (red: TRITC and blue: DAPI) in the cortices and hippocampi of the APP/PS1 and Adipo^−/−^ mice respectively. The data are indicated as the ± SEM for *n* = 5 mice per group, and the number of independent experiments = 3. Magnification: 40X. Scale bar = 50 μm. Significance = ***p < 0.001; ###*p* < 0.001; One-way ANOVA followed by Turkey’s post hoc test. (**g-j)** Histograms present ELISA and immunoassay results for the AdipoR1 and AMPK levels respectively, in vivo brain homogenates of APP/PS1 and Adipo^−/−^ mice. ELISA and immunoassay data are expressed as the means ± SEM for the indicated proteins  in vivo (n = 8 mice/group), and the number of independent experiments = 3. Significance = ***p < 0.001; ###*p* < 0.001; One-way ANOVA followed by Turkey’s post hoc test

**Fig. 2:**
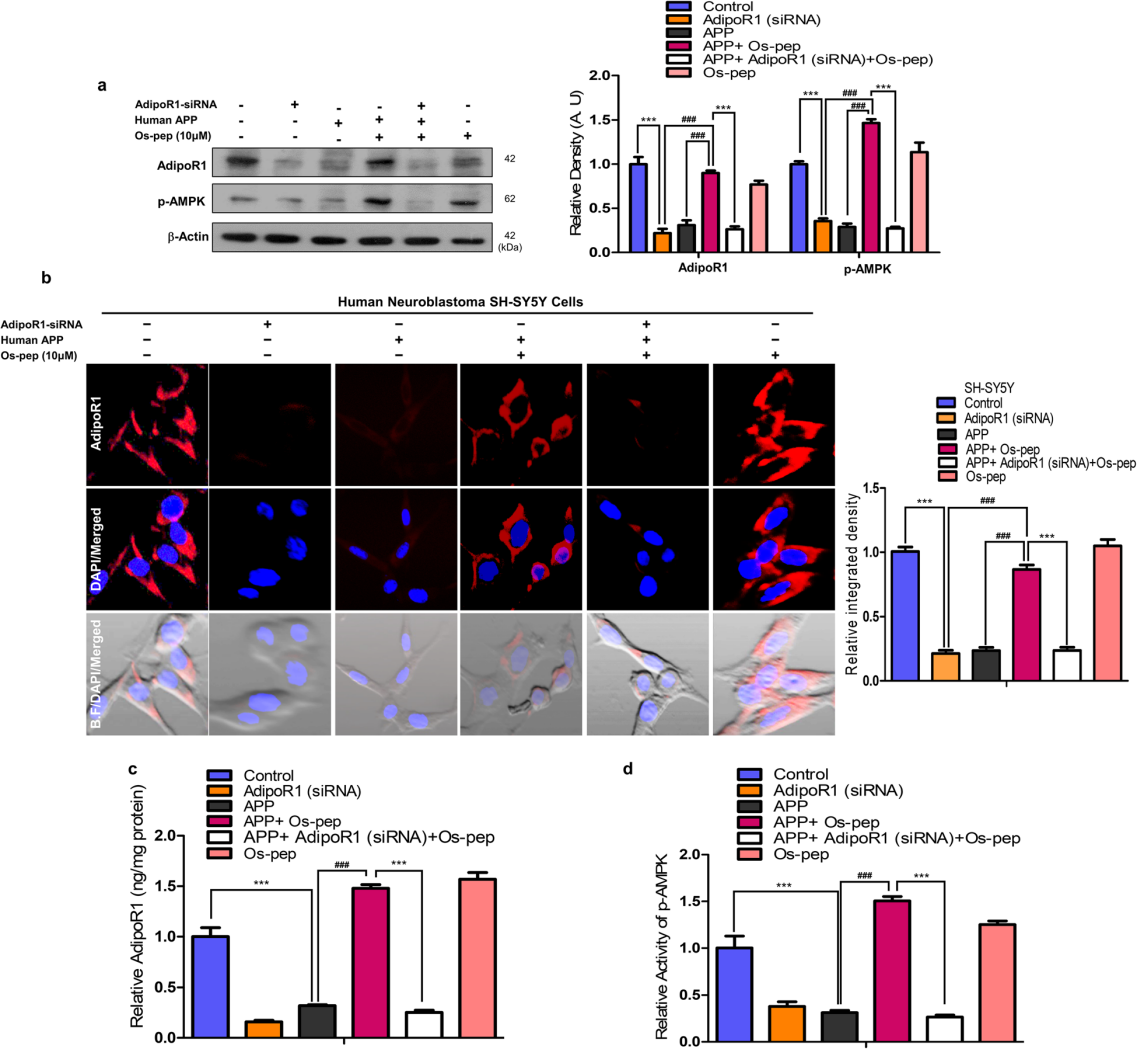
Os-pep stimulated AdipoR1/p-AMPK signaling in in vitro AD models. (**a)** Immunoblotting and quantification of AdipoR1 and p-AMPK levels in human APPswe/ind-overexpressed SH-SY5Y cells transfected with the AdipoR1 siRNA and treated with Os-pep (10 μM). The data are expressed as the means ± SEM for the indicated proteins in vitro (n = 5/group), and the number of independent experiments = 3. Significance = ***p < 0.001; ###*p* < 0.001; Two-way ANOVA followed by Bonferroni’s multiple comparisons test (**b)** Immunofluorescence reactivity for AdipoR1 (Red: TRITC and Blue: DAPI, Brightfield; B.F.) in human APPswe/ind-overexpressed SH-SY5Y cells transfected with the AdipoR1 siRNA and treated with Os-pep (10 μM). Magnified 40X. Scale bar = 20 μm. The data are indicated as the ± SEM for n = 5 images per group, and the number of independent experiments = 3. Significance = ***p < 0.001; ###*p* < 0.001; Two-way ANOVA followed by Bonferroni’s multiple comparisons test **(c, d)** Histograms present ELISA and immunoassay results for the AdipoR1 and AMPK levels respectively, in vitro cell lysates of human APPswe/ind-overexpressed SH-SY5Y cells expressing the AdipoR1 siRNA in the presence or absence of the Os-pep dosage (10 μM) for 12 h. The data are expressed as the means ± SEM for the indicated proteins in vitro (n = 5/group), and the number of independent experiments = 3. Significance = ***p < 0.001; ###*p* < 0.001; Two-way ANOVA followed by Bonferroni’s multiple comparisons test

Remarkable studies have reported reduced neuronal AdipoR1 levels in *in vitro* and *in vivo* AD models [21, 39, 40]. Furthermore, our study and other interesting and compelling studies reported that neuronal AdipoR1 suppression is associated with metabolic stress, which leads to AD pathology and memory impairment [13–15, 21, 46, 47]. Therefore, we aimed to investigate neuronal AdipoR1 and its downstream effector p-AMPK, a key energy sensor marker involved in metabolic stress, in human APP Swedish (swe)/Indiana (ind)-overexpressing SH-SY5Y cells and AβO-exposed SH-SY5Y cells *in vitro* AD models. Consistently, we observed reduced AdipoR1/p-AMPK levels in AβO-exposed and human APPswe/ind-overexpressing SH-SY5Y cells. We observed that the use of the optimized concentration (10 μM) of Os-pep significantly increased neuronal AdipoR1/p-AMPK signaling in AβO-treated and human APPswe/ind-overexpressing SH-SY5Y cells (Fig. S1a,b). Next, through immunofluorescence staining, we observed that Os-pep (10 μM) significantly increased neuronal AdipoR1 immunoreactivity in AβO-exposed SH-SY5Y cells (Fig. S1c). Furthermore, to confirm the importance of AdipoR1 as a potential target for AD, we analyzed human brain samples from aged healthy and AD patients via confocal microscopy and interestingly found reduced AdipoR1 levels in the cortical and hippocampal regions of AD human brains compared to those in aged healthy human brains (Fig. 1a). Next, we optimized the Os-pep dosage regimen for *in vivo* studies and administered Os-pep (5 μg/g, i.p., on alternating days for 45 days) to mice subjected to AβO injection. Notably, Os-pep significantly increased AdipoR1/p-AMPK signaling in the AβO-injected mice compared with that in the AβO-injected mice without Os-pep treatment. We also noted that chronic Os-pep treatment alone (5 μg/g, i.p., on alternating days for 45 days) did not induce any undesirable effects even though it provided beneficial effects by activating AdipoR1/−pAMPK signaling (Fig. 1b). Next, to extend our approach in a more rigorous AD *in vivo* model, we administered Os-pep (5 μg/g, i.p., on alternating days for 45 days) to APP/PS1 mice, which is an established transgenic AD model. Interestingly, we found that Os-pep remarkably increased AdipoR1/p-AMPK signaling in the APP/PS1 group compared to that in the Veh-treated APP/PS1 mice (Fig. 1c). Furthermore, it has been recently shown that Adipo^−/−^ mice exhibit aberrant AdipoR1/AMPK signaling, neuronal insulin resistance and AD pathology [14–16]. Here, we also used Adipo^−/−^ mice to verify our hypothesis and the adiponectin-mimetic activity of Os-pep. Notably, we observed reduced levels of AdipoR1/AMPK in the Veh-injected Adipo^−/−^ mice. However, the administration of the Os-pep dosage (5 μg/g, i.p., on alternating days for 45 days) to Adipo^−/−^ mice significantly increased AdipoR1/AMPK levels compared to those in the group without Os-pep treatment (Fig. 1d). To validate the Western blotting results, we performed confocal microscopy. We observed that Os-pep administration to APP/PS1 and Adipo^−/−^ mice significantly increased AdipoR1 immunofluorescence reactivity in brain tissues (Fig. 1e,f). As a supplement to immunoblotting and confocal microscopy, we performed AdipoR1 ELISA and AMPK immunoassays using mouse brain homogenates. Based on the AdipoR1 ELISA and AMPK assay results, AdipoR1 and AMPK levels were significantly lower in the Veh-injected APP/PS1 and Adipo^−/−^ mice than in the control WT Veh-treated mice. Notably, Os-pep remarkably enhanced AdipoR1/AMPK expression in APP/PS1 and Adipo^−/−^ mice compared to those in Veh-injected APP/PS1 and Adipo^−/−^ mice (Fig. 1g-j). Next, to determine the mechanism, we treated APPswe/ind-overexpressing SH-SY5Y cells with Os-pep (10 μM) with or without AdipoR1 siRNA. Interestingly, we found a reduction in AdipoR1 in the APPswe/ind-overexpressing SH-SY5Y cells, which was reversed by Os-pep, leading to a significant increase in AdipoR1; however, the levels of AdipoR1 and its downstream target p-AMPK were further decreased in the APPswe/ind-overexpressing SH-SY5Y cells upon AdipoR1 knockdown (Fig. 2a, b). We further performed AdipoR1 ELISA and AMPK immunoassays using the cell lysate samples. Based on the AdipoR1 ELISA and AMPK assay results, AdipoR1 and AMPK expression levels were significantly reduced in neuronal SH-SY5Y cells overexpressing APPswe/ind. Os-pep remarkably enhanced AdipoR1/AMPK expression in SH-SY5Y cells overexpressing APPswe/ind. However, when AdipoR1 expression was knocked down in SH-SY5Y cells, the interaction of Os-pep with AdipoR1 decreased, and Os-pep did not promote the stimulation of AdipoR1/AMPK signaling, validating that Os-pep acts via AdipoR1 and activates AMPK signaling (Fig. 2c, d). Based on results from previous studies [43–45] and our rigorous *in vivo* and *in vitro* results described above, Os-pep functions as an adiponectin mimetic and activates AdipoR1/AMPK signaling.

### Os-pep rescued neuronal insulin resistance and activated downstream insulin signaling via AdipoR1/AMPK signaling

**Fig. 3:**
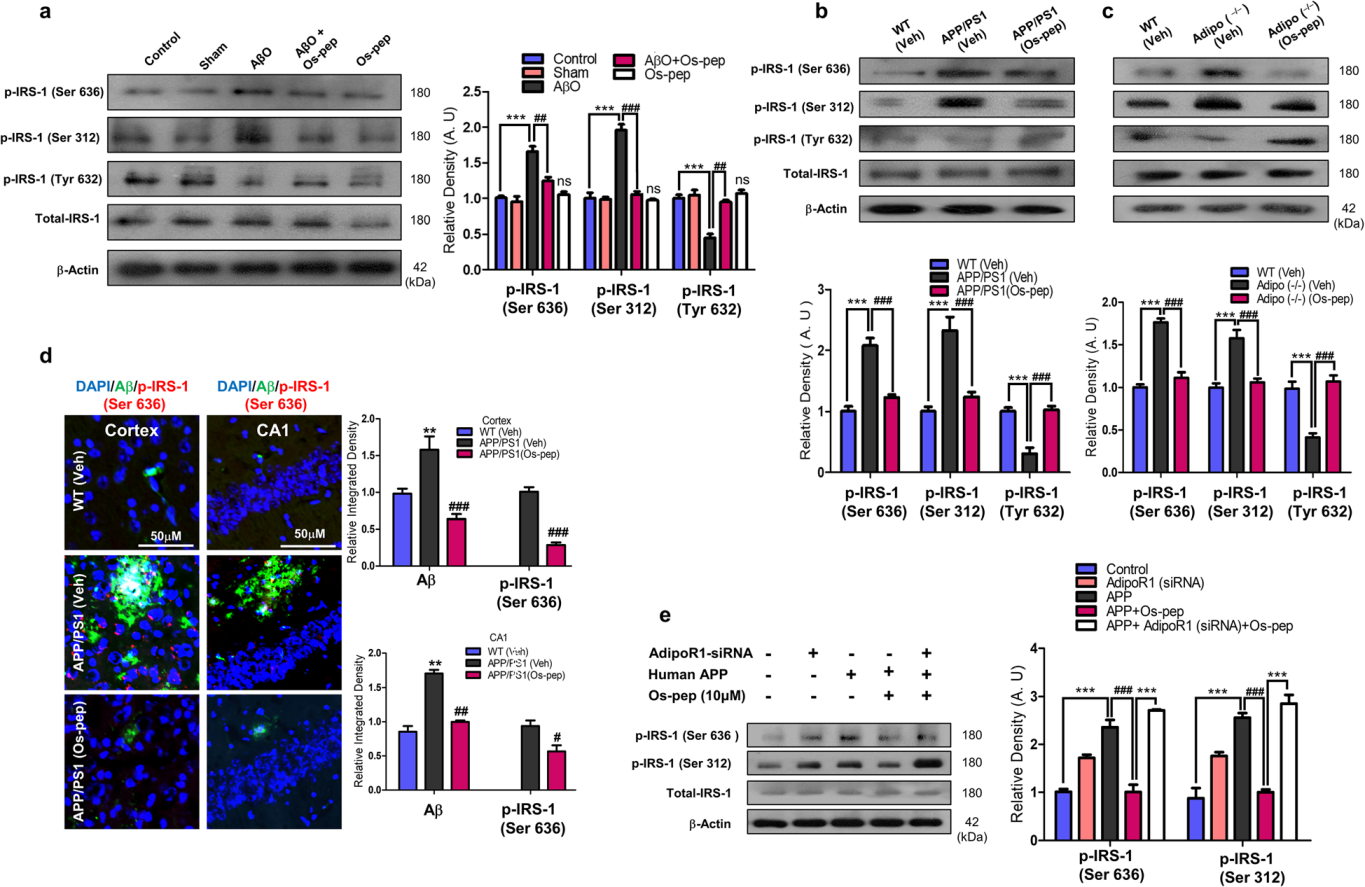
Os-pep rescued neuronal insulin resistance via AdipoR1/AMPK signaling in vitro and in vivo AD models and Adipo^−/−^ mice. **(a-c)** Immunoblotting and quantification of p-IRS-1 (Ser 636)/ total-IRS-1 and p-IRS-1 (Ser 312)/total-RS-1 levels in the AβO, APP/PS1 and Adipo^−/−^ mice. The data are expressed as the means ± SEM for the indicated proteins in vivo (n = 8 mice/group) and the number of independent experiments = 3. Significance = **p < 0.01, ***p < 0.001; ###*p* < 0.001; One-way ANOVA followed by Turkey’s post hoc test. ns, indicated that there is no significant difference among the control, sham and Os-pep treated groups **(d)** Double immunofluorescence staining for Aβ (FITC, green) and p-IRS-1 (Ser 636) (TRITC, red) in the cortex and hippocampus of APP/PS1 mice. The data are expressed as the means ± SEM for *n* = 5 mice/group, and the number of independent experiments = 3. Magnification 40x and magnified 40X. Scale bar = 50 μm and 20 μm respectively. Significance = **p < 0.01, ***p < 0.001; #*p* < 0.05, ##*p* < 0.01, ###*p* < 0.001; One-way ANOVA followed by Turkey’s post hoc test. (**e)** Immunoblotting and quantification of p-IRS-1 (Ser 636)/total-IRS-1 and p-IRS-1 (Ser 312)/total-IRS-1 levels in the human APPswe/ind-overexpressed SH-SY5Y cells transfected with the AdipoR1 siRNA and treated with Os-pep (10 μM). The data are expressed as the means ± SEM for the indicated proteins in vitro (n = 5/group), and the number of independent experiments = 3. Significance = ***p < 0.001; ###*p* < 0.001; Two-way ANOVA followed by Bonferroni’s multiple comparisons test

**Fig. 4:**
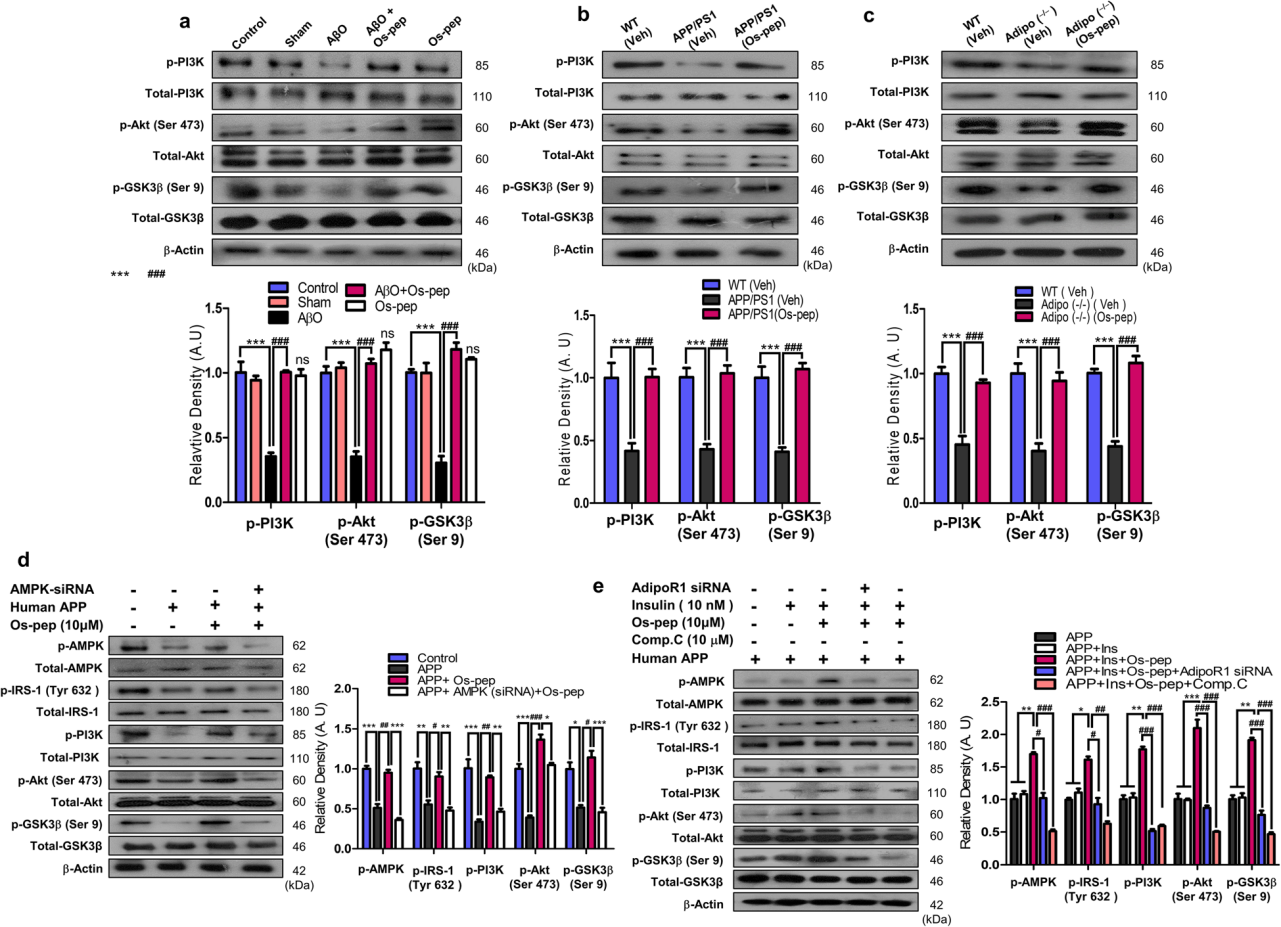
Os-pep activated downstream insulin signaling via AdipoR1/AMPK signaling in vitro and in vivo AD models and Adipo^−/−^ mice. **(a-c)** Immunoblotting and quantification of p-PI3K/total-PI3K, p-Akt/total-Akt and p-GSK3β (Ser 9)/total-GSK3β levels in the AβO-treated, APP/PS1 and Adipo^−/−^ mice. The data are expressed as the means ± SEM for the indicated proteins in vivo (n = 8 mice/group) and the number of independent experiments = 3. Significance = ***p < 0.001; ###*p* < 0.001; One-way ANOVA followed by Turkey’s post hoc test. ns, indicated that there is no significant difference among the control, sham and Os-pep treated groups (**d)** Immunoblotting and quantification of p-AMPK/total-AMPK, p-IRS-1 (Tyr632)/total-IRS-1, p-PI3K/total-PI3K, p-Akt/total-Akt and p-GSK3β (Ser 9)/total-GSK3β levels in APPswe/ind-overexpressed SH-SY5Y cells transfected with the AMPK siRNA and treated with Os-pep (10 μM). The data are expressed as the means ± SEM for the indicated proteins in vitro (n = 5/group), and the number of independent experiments = 3. Significance = *p < 0.05, **p < 0.01 ***p < 0.001; #*p* < 0.05, ##*p* < 0.01, ###*p* < 0.001; Two-way ANOVA followed by Bonferroni’s multiple comparisons test **(e)** Immunoblotting and quantification of p-AMPK/total-AMPK, p-IRS-1 (Tyr 632)/total-IRS-1, p-PI3K/total-PI3K, p-Akt/total-Akt and p-GSK3β (Ser 9)/total-GSK3β levels in APPswe/ind-overexpressed SH-SY5Y cells exposed to a high insulin (1 μM) concentration (SH-SY5YIR), low insulin concentration (10 nM), Os-pep (10 μM) and Comp. C (10 μM) and transfected with the AdipoR1 siRNA. The data are expressed as the means ± SEM for the indicated proteins in vitro (n = 5/group), and the number of independent experiments = 3. Significance = *p < 0.05, **p < 0.01 ***p < 0.001; #*p* < 0.05, ##*p* < 0.01, ###*p* < 0.001; Two-way ANOVA followed by Bonferroni’s multiple comparisons test

Numerous studies have reported the role of aberrant neuronal insulin resistance in brain metabolism, which is associated with AD. Neuronal insulin resistance instigated by hyperphosphorylation of IRS-1 at the Ser^636/312/307^ residues and downregulation of p-IRS-1^Tyr 632^ has been associated with AD pathology and reported in Aβ and APP/PS1 mouse AD models as well as HFD and Adipo−/− mice [8, 9, 14–16, 21, 48]. Concomitantly, we also observed increased levels of p-IRS-1 Ser^636/312/307^ and reduced levels of p-IRS-1^Tyr 632^ in the in the Veh-treated AβO-treated, APP/PS1 and Adipo−/− and HFD mice. Intriguingly, Os-pep treatment (5 μg/g, i.p., on alternating days for 45 days) reversed these effects, significantly reducing the increase in p-IRS-1^Ser636/312^ levels and significantly increasing the p-IRS-1^Tyr 632^ levels in the AβO-treated, APP/PS1, Adipo^−/−^ and HFD mice compared with those in the Veh-treated AβO-treated, APP/PS1, Adipo^−/−^ and HFD mice, respectively (Fig. 3a-c, Fig. S2). Furthermore, double immunofluorescence images indicated that Os-pep reduced the colocalization of the immunofluorescence reactivity of Aβ and p-IRS-1^Ser636^ in the APP/PS1 mice (Fig. 3d). We intended to verify whether Os-pep regulates neuronal insulin resistance through AdipoR1. Therefore, we used AdipoR1 siRNA to determine the mechanism by which Os-pep reduced neuronal insulin resistance in AD models. When we knocked down AdipoR1, Os-pep did not downregulate p-IRS-1^Ser636/312^ in the APPswe/ind-overexpressing SH-SY5Y cells (Fig. 3e). Furthermore, Os-pep treatment (5 μg/g, i.p., on alternating days for 45 days) regulated the levels of various biochemical parameters associated with metabolic disorders and AD in the serum of both APP/PS1, HFD and Adipo^−/−^ mice (Fig. S3a-d; Fig. S4a-d). We also observed a smaller fold-increase in the body weight of the Os-pep-treated APP/PS1 HFD and Adipo^−/−^ mice compared with that of the Veh-treated APP/PS1, HFD and Adipo^−/−^ mice (Fig. 3e; Fig. S4e). Moreover, Western blotting was performed to assess the role of Os-pep in the important p-PI3K/Akt/GSK3β (Ser 9) pathway, which is downstream of insulin signaling, in the AβO-treated, APP/PS1 and Adipo^−/−^ mice. Os-pep treatment (5 μg/g, i.p., on alternating days for 45 days) reversed the reduction of p-PI3K/Akt/GSK3β (Ser 9) levels in the AβO-treated, APP/PS1 and Adipo^−/−^ mice compared with that in the Veh-treated AβO-treated, APP/PS1 and Adipo^−/−^ mice, respectively (Fig. 4a-c). Several compelling studies have shown that activation of AMPK plays a vital role in the activation of downstream insulin signaling [9–11, 14–16]. As we showed that Os-pep activated AMPK signaling, we used AMPK siRNA to determine the mechanism by which Os-pep regulated downstream insulin signaling in the AD model. Os-pep treatment did not upregulate the p-IRS-1^Tyr 632^ and p-PI3K/Akt/GSK3β (Ser 9) pathways in human APPswe/ind-transfected neuronal SH-SY5Y cells with AMPK knockdown using AMPK siRNA, suggesting that Os-pep treatment plays a key role in triggering AdipoR1 activation and downstream p-AMPK signaling and, consequently, in the regulation of insulin signaling in AD models (Fig. 4d). Human APPswe/ind-transfected neuronal SH-SY5Y cells with insulin resistance were exposed to a high insulin concentration (1 μM) (SH-SY5YIR) to determine the mechanism underlying the insulin-sensitizing activity of Os-pep. The levels of p-AMPK, p-IRS-1^Tyr 632^ and p-PI3K/Akt/GSK3β (Ser 9) were not elevated after exposure to a low insulin (10 nmol/L) concentration. However, Os-pep (10 μM) significantly activated the p-IRS-1^Tyr 632^ and p-PI3K/Akt/GSK3β (Ser 9) pathways through the activation of p-AMPK, indicating that Os-pep reduced insulin resistance via an AdipoR1/p-AMPK-dependent mechanism (Fig. 4e). Moreover, AdipoR1 siRNA and the AMPK inhibitor compound C (Comp. C) (10 μM) inhibited the insulin-sensitizing effect of Os-pep. Based on these results, Os-pep induced insulin sensitivity by increasing the levels of p-IRS-1^Tyr 632^ and p-PI3K/Akt/GSK3β (Ser 9) through an AdipoR1/AMPK-dependent mechanism to exert its beneficial effects (Fig. 4e). Collectively, the results show that Os-pep reduced neuronal insulin resistance and activated insulin signaling in the brain by stimulating AdipoR1/AMPK signaling to exert beneficial effects on AD pathology.

### Os-pep via AdipoR1 restored synaptic functions in mice with AD-mimicking pathological conditions and in Adipo^−/−^ mice

**Fig. 5:**
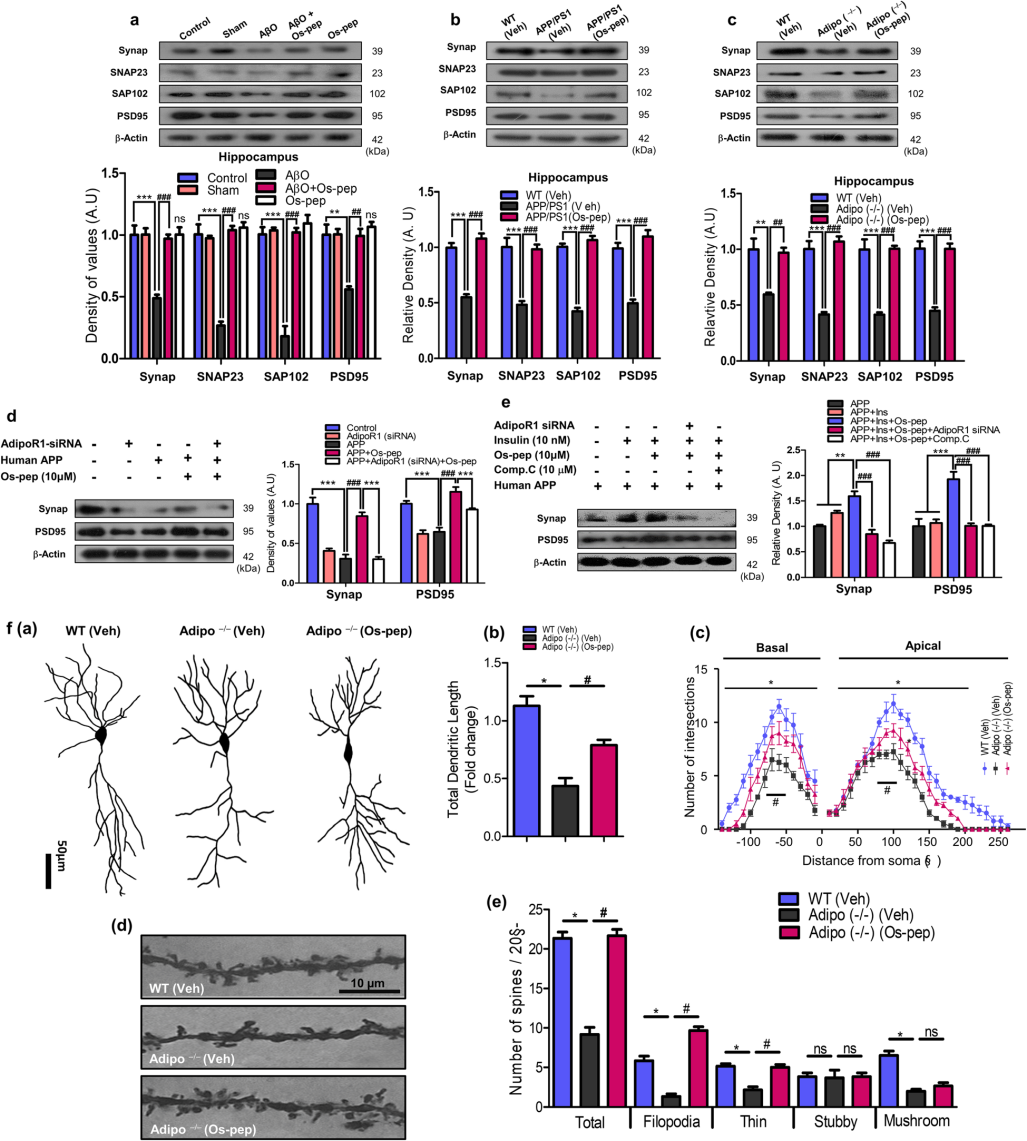
Os-pep restored synaptic functions in mice and in vitro with AD-mimicking pathological conditions and Adipo^−/−^ mice. **(a-c)** Immunoblotting and quantification of Synap, SNAP-23, SAP102 and PSD95 levels in the AβO, APP/PS1 and Adipo^−/−^ mice respectively. The data are expressed as the means ± SEM for the indicated proteins in vivo (*n* = 8 mice/group) and the number of independent experiments = 3. Significance = ***p < 0.001; ###*p* < 0.001; One-way ANOVA followed by Turkey’s post hoc test. ns, indicated that there is no significant difference among the control, sham and Os-pep treated groups (**d)** Immunoblotting and quantification of the Synap and PSD95 levels in APPswe/ind-overexpressed SH-SY5Y cells transfected with the AdipoR1 siRNA and treated with Os-pep (10 μM). The data are expressed as the means ± SEM for the indicated proteins in vitro (n = 5/group), and the number of independent experiments = 3. Significance = ***p < 0.001; ###*p* < 0.001; Two-way ANOVA followed by Bonferroni’s multiple comparisons test **(e)** Immunoblotting and quantification of the Synap and PSD95 levels in APPswe/ind-overexpressed SH-SY5YIR cells exposed to a high insulin (1 μM) concentration, a low insulin concentration (10 nM), Os-pep (10 μM) and Comp. C (10 μM) and transfected with the AdipoR1 siRNA. The data are expressed as the means ± SEM for the indicated proteins in vitro (n = 5/group), and the number of independent experiments = 3. Significance = ***p < 0.01, **p < 0.01; ###*p* < 0.001; Two-way ANOVA followed by Bonferroni’s multiple comparisons test. (**f)** Os-pep regulated dendritic complexity and spine density in the Adipo^−/−^ mice. (**f, panel** (**a**)) Indicated images were the example of the reconstructed hippocampal CA1 region of pyramidal neurons in the WT (Veh), Adipo^−/−^ and Os-pep treated Adipo^−/−^ (Os-pep). (**f, panel** (**b**)) Representative histogram indicated total dendritic length (sum of basal and apical dendrite length) in the hippocampal CA1 region of the mice. (**f, panel** (**c**)) Representative histogram indicated dendritic complexity via using the sholl analysis of reconstructed pyramidal neurons. (**f, panel** (**d**)) Representative images of digitalized hippocampal CA1 pyramidal dendrites from the secondary branches. (**f, panel** (**e**)) Representative histograms indicate the dendritic spines density; including the total number of spines, the number of filopodia-like, thin, mushroom and stubby spines in the hippocampal CA1 region of brain mice. The data are shown as the mean ± SEM of 45 pyramidal neurons and 300 dendritic segments per groups and the number of independent experiments = 3. Significance = *p < 0.05; #*p* < 0.05; One-way ANOVA followed by Turkey’s post hoc test

To investigate the effect of Os-pep on synaptic function, we assessed memory-associated pre- and postsynaptic protein markers. Os-pep (5 μg/g, i.p., on alternating days for 45 days) remarkably increased pre- and postsynaptic protein levels in AD (AβO-treated, APP/PS1) and Adipo^−/−^ mice (Fig. 5a-c). Os-pep (5 μg/g, i.p., on alternating days for 45 days) administration alone in WT normal mice did not have any detrimental effects on the pre- and postsynaptic markers, which provides a proof of principle that chronic Os-pep administration has no harmful effects (Fig. 5a). Furthermore, to assess mechanistically the roles of AdipoR1/AMPK signaling and neuronal insulin resistance, we analyzed the two main pre- and postsynaptic protein markers (synaptophysin (Synap) and postsynaptic density protein (PSD95), respectively) in human neuronal SH-SY5Y cells overexpressing APPswe/ind and expressing AdipoR1 siRNA and in APPswe/ind-overexpressing SH-SY5YIR cells exposed to AdipoR1 siRNA and treated with Comp. C (10 μM). Importantly, Os-pep restored and significantly increased Synap and PSD95 expression in APPswe/ind-overexpressing SH-SY5Y cells; however, Os-pep did not restore or increase Synap and PSD95 expression in APPswe/ind-overexpressing SH-SY5Y cells exposed to AdipoR1 siRNA (Fig. 5d). Interestingly, when SH-SY5YIR cells overexpressing APPswe/ind expressed AdipoR1 siRNA and were treated with Comp. C, Os-pep did not increase the levels of Synap and PSD-95 (Fig. 5e).

Next, to test the effect of Os-pep on dendritic complexity and spine density, we designed separate cohort studies and performed Golgi staining and analyses. In Fig. 5f, Panel (a), and Fig. S5a, three representative pyramidal neurons are shown, and a remarkable decrease in the total dendritic length was found in Adipo^−/−^ and AβO mice. Os-pep (5 μg/g, i.p., on alternating days for 45 days) significantly increased the total dendritic length (Fig. 5f, Panel (a, b); Fig. S5a, b). Likewise, administration of Os-pep to Adipo^−/−^ and AβO mice remarkably enhanced the complexity of dendrites in the apex and base region (Fig. 5f, Panel (c); Fig. S5c). Furthermore, we observed significantly reduced spine numbers in the CA1 region of the hippocampus in the Adipo^−/−^ and AβO mice (Fig. f, Panel (d); Fig. S5d). Notably, a reduced number of filopodia-like spines were observed in the Adipo^−/−^ and AβO mice (Fig. 5f, Panel (e); Fig. S5e); nevertheless, no significant change was observed in the numbers of thin, stubby and mushroom-like spines. Interestingly, Os-pep administration to Adipo^−/−^ and AβO mice significantly increased the total number of spines and the number of filopodia-like spines. These results revealed that Os-pep had beneficial effects on dendrites and spine density in Adipo^−/−^ and AD mice.

**Fig. 6:**
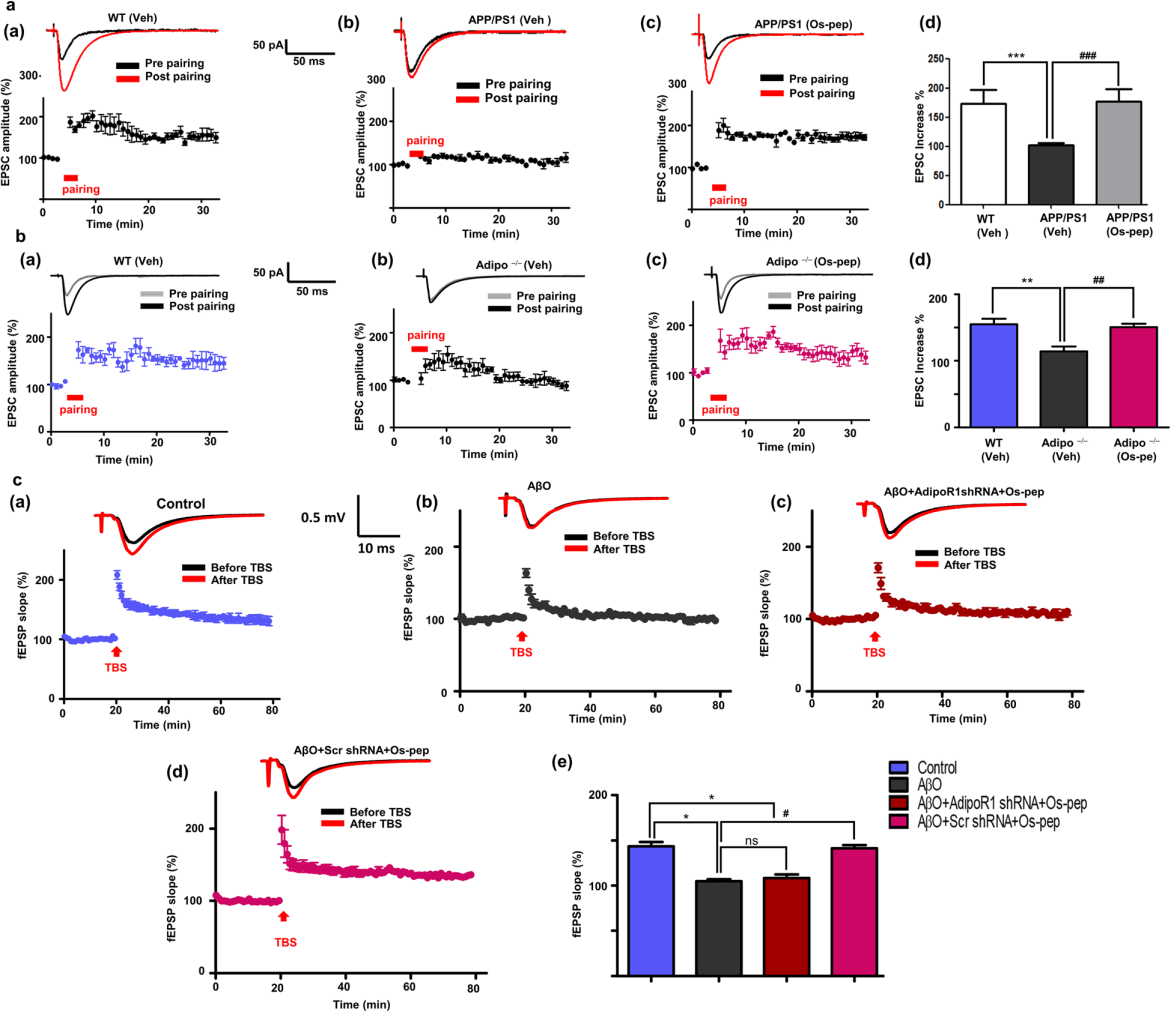
Os-pep restores LTP ability in the APP/PS1, Adipo^−/−^ and AβO-treated mice via AdipoR1 dependent manner. **(a, panels (a-c); b, panels (a-c**)) Upper trace, a representative EPSC trace before and after LTP was induced by paired stimuli in the CA1 regions of the hippocampi of Veh-injected WT or Veh-injected APP/PS1 and Adipo^−/−^ mice respectively. Lower trace, an average time course of EPSCs before and after LTP was induced by paired stimuli in the CA1 regions of the hippocampi of Veh-injected WT, APP/PS1 and Adipo^−/−^ mice, as well as Os-pep-treated APP/PS1 and Adipo^−/−^ mice. (**a, panel (d); b, panel (d**)) A summary of the effect of the Os-pep treatment on LTP induction in APP/PS1 and Adipo^−/−^ mice respectively. (**c, panels (a-d**)) Upper trace, a representative EPSC trace before and after LTP was induced by TBS stimuli in the CA1 regions of the hippocampi of Veh-injected WT or Veh-injected AβO. Lower trace, an average time course of EPSCs before and after LTP was induced by paired stimuli in the CA1 regions of the hippocampi of Veh-injected WT or Veh-injected AβO mice, as well as Os-pep-treated AβO-injected mice subjected to scramble shRNA and shRNA mediating silencing of AdipoR1 gene. **(c, panel (e**)) A summary of the effect of the Os-pep dosage on LTP induction in Veh-injected WT or Veh-injected AβO mice, as well as Os-pep-treated AβO mice subjected to scramble and shRNA mediating silencing of AdipoR1 gene. The data are expressed as the means ± SEM for n = 5–6 mice per group and the number of independent experiments = 3. Significance = *p < 0.05, **p < 0.01 ***p < 0.001; #*p* < 0.05, ##*p* < 0.01, ###*p* < 0.001; One-way ANOVA followed by Turkey’s post hoc test

We also investigated whether Os-pep modulated synaptic function and plasticity using electrophysiology. In Fig. 6a, panels (a,b); b, panels (a,b), the results indicated that LTP was remarkably reduced in slices of the CA1 region of the hippocampus in APP/PS1 and Adipo^−/−^ mice. Os-pep improved LTP in the APP/PS1 and Adipo^−/−^ mice (Fig. 6a, panels (b,c); b panels (a,b)). The percentage increase in EPSCs induced by paired stimuli is shown for the APP/PS1 and Adipo^−/−^ mice (Fig. 6a, panel (d); b, panel (d)). Furthermore, to validate the mechanism of the effect of Os-pep on LTP improvements, we determined whether these improvements are AdipoR1 dependent or not. We designed a separate group of studies in which we used AβO-treated (subjected to treatment with scramble and functional AdipoR1 shRNA) AD mice. As shown in Fig. 6c, panels (a, b), LTP was significantly attenuated in AβO mice. The suppression of LTP was almost completely rescued to a level comparable to that of normal controls by Os-pep in scramble shRNA-treated AβO mice (Fig. 6c, panel (d)). Nevertheless, the potentiating effect of Os-pep on LTP was significantly attenuated by shRNA-mediated silencing of the AdipoR1 gene (Fig. 6c, panels (c, e)), strongly implying the contribution of AdipoR1 to Os-pep-induced restoration of LTP. These intriguing results strongly suggest that Os-pep increases pre- and postsynaptic protein markers as well as enhances LTP in preclinical animal models via activation of AdipoR1 signaling.

### The Os-pep dosage regimen improved learning and memory functions via AdipoR1 in AD and Adipo^−/−^ mice

**Fig. 7:**
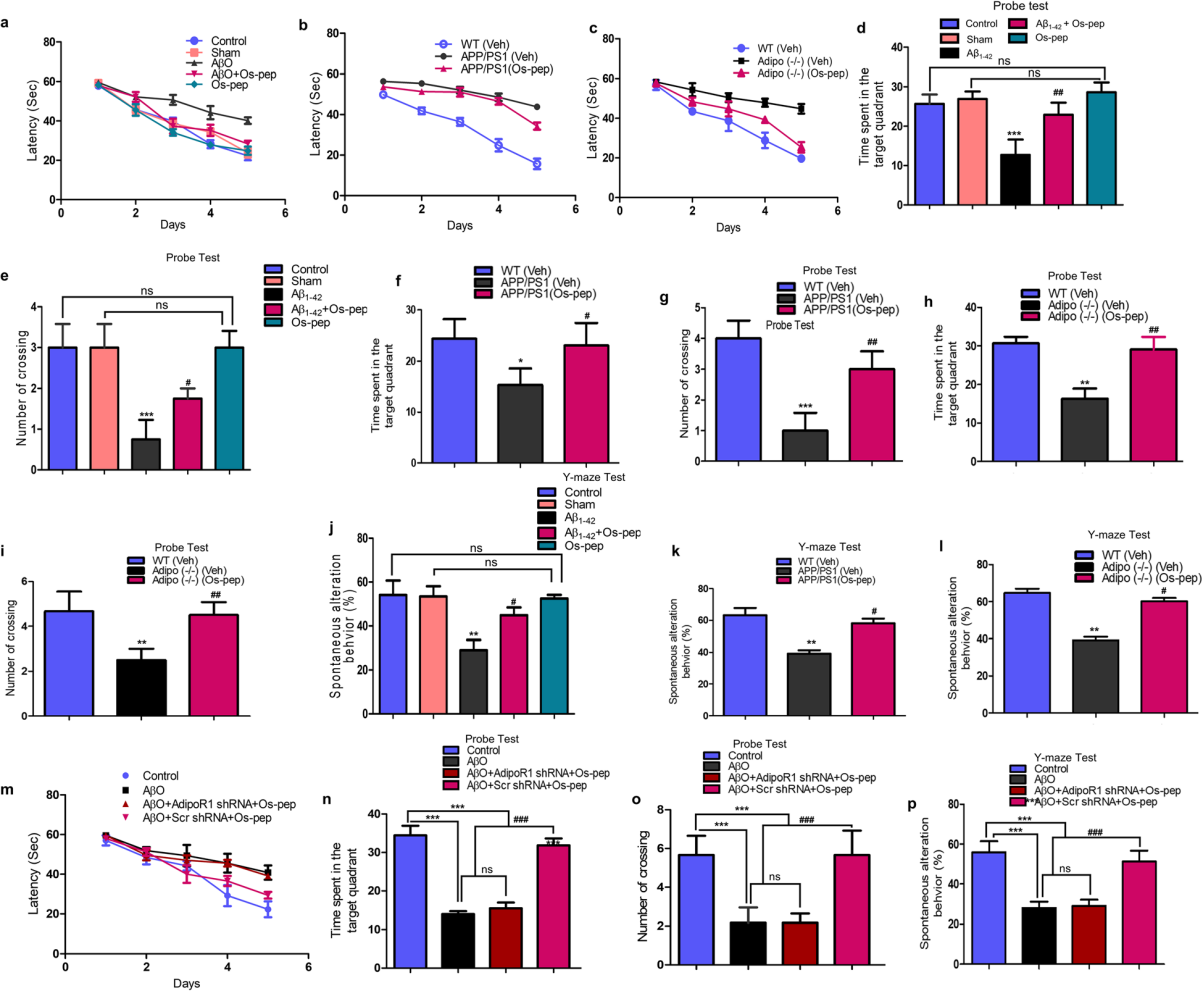
Os-pep via AdipoR1 dependently improved the learning and memory of AD, Adipo^−/−^ and AdipoR1 shRNA mice. MWM and Y-maze assays were used to assess the spatial working memory and cognitive functions in the AD mouse models and in the Adipo^−/−^ mice. Thirteen mice from each group were used for the behavioral evaluation. **(a-c)** The mean escape latency (sec) to reach the hidden platform during training of the AβO-treated, APP/PS1 and Adipo^−/−^ mice. (**d-i)** The time spent in the target quadrant and number of platform crossings during the probe test of the AβO-treated, APP/PS1 and Adipo^−/−^ mice. **(j-l)** Histograms present the percentage of spontaneous alternation behaviors by the AβO-treated, APP/PS1 and Adipo^−/−^ mice respectively during the Y-maze test. **(m)** The mean escape latency (sec) to reach the hidden platform during training of the AβO-treated (subjected to scramble and functional AdipoR1 shRNA). (**n, o)** The time spent in the target quadrant and number of platform crossings during the probe test of the AβO-treated (subjected to scramble and functional AdipoR1 shRNA). **(p)** Histograms present the percentage of spontaneous alternation behaviors by the AβO-treated (subjected to scramble and functional AdipoR1 shRNA) during the Y-maze test. The escape latencies in the behavioral tests were analyzed using a two-way ANOVA, with training days as the repeated measurements. Graphs show the means ± SEM for the mice (13 mice per group) and the number of independent experiments = 3. Significance = *p < 0.05, **p < 0.01 ***p < 0.001; #*p* < 0.05, ##*p* < 0.01, ###*p* < 0.001; One-way ANOVA followed by Turkey’s post hoc test. ns, indicated that there is no significant difference among the control, sham and Os-pep treated groups

For the first time in this study, to the best of our knowledge, the small 9-amino acid Os-pep, which is considered an adiponectin-mimetic peptide, was evaluated for its effect on memory and learning functions in well-established AβO-treated and APP/PS1 transgenic mouse models of memory deficits and AD as well as in Adipo^−/−^ mice; it was recently reported that Adipo^−/−^ mice have deficits in learning and memory functions [14, 16]. Following Os-pep regimen (5 μg/g, i.p., on alternating days for 45 days) completion, the mice were evaluated in behavioral studies with the MWM and Y-maze tests. In the MWM tests, we initially observed that Os-pep enhanced and restored the learning/memory functions of the AβO-injected and APP/PS1 mice as well as the Adipo^−/−^ mice, as revealed by the decrease in the latency to reach the submerged platform in the training session (Fig. 7 a-c). Furthermore, the probe test results indicated that Os-pep significantly improved learning and memory functions, as more time was spent in the target quadrant, and there was a significantly increased number of platform crossings in the AβO-injected, APP/PS1 and Adipo^−/−^ groups (Fig. 7d-i). Additionally, the swimming speeds in the training session and the final day prior to the probe test were not significantly different among all groups (Fig. S6a, panels (a-i)). Next, the Y-maze results indicated that Os-pep significantly increased the percentage of spontaneous alternation behaviors in the AβO-injected, APP/PS1 and Adipo^−/−^ mice (Fig. 7j-l**)**, indicating that Os-pep improved spatial working memory functions.

To determine the mechanistic influence of Os-pep on memory functions, we designed a separate group of studies (AβO-injected mice exposed to scramble shRNA and shRNA silencing of the AdipoR1 gene). The measurement of latency during training, the probe test and the spontaneous alternation behavior percentage indicated that Os-pep did not improve spatial learning/memory functions in the AβO-injected mice exposed to shRNA silencing of the AdipoR1 gene compared to those in animals that received the scramble shRNA (Fig. 7m-p). Similarly, the mouse swimming speeds in the training session and on the 7th day prior to the probe test were not significantly different among all groups (Fig. S6b, panels (a-c)). Based on these observations, Os-pep chronic administration (5 μg/g, i.p., on alternating days for 45 days) rescued the memory deficits associated with aberrant neuronal metabolic-associated AD pathology via activation of AdipoR1-dependent processes, and these results corroborate and validate the above *in vivo* and *in vitro* biochemical and immunohistological results.

### Acute bolus dosages and chronic optimized dosages have no major adverse effects on mice

To optimize and analyze the toxicity and effective dose of Os-pep, we performed a separate group of studies in order to assess signs of systemic toxicity, such as head tilt, tremor, changes in physical activity and squinting, for 1 week in normal WT mice injected i.p. with bolus doses of Os-pep (10, 20, 30, 40 and 50 mg/kg) to investigate the safety profile of bolus doses of Os-pep. We observed a profile indicating that Os-pep was safe, and no toxicity was observed in any of the mice treated with bolus doses (data not shown).

When Os-pep was administered chronically at an optimized dosage (5 μg/g, i.p., on alternating days for 45 days) to all mice, the immunoblotting results showed that Os-pep didn’t induce any harmful effects on any cellular signaling (e.g. AdipoR1/AMPK, insulin resistance and downstream insulin signaling) in brain as well as synaptic functions.

In addition to the observations and results described above (Fig. S3a-d; Fig. S4a-d), Os-pep regulated the biochemical parameters associated with metabolic disorder and AD in the serum of APP/PS1, HFD and Adipo^−/−^ mice. These results suggested that Os-pep did not induce liver or kidney dysfunction. All the observations of mice treated with bolus doses and chronic dosage regimens proved that Os-pep has no observable side effects in animal models and acts as a safe adiponectin-mimetic peptide. However, further biochemical and molecular-based studies of the peripheral organs will be assessed for the acute and chronic administration of Os-pep.

### Neuroprotective and pharmacokinetic profile of Os-pep

**Fig. 8:**
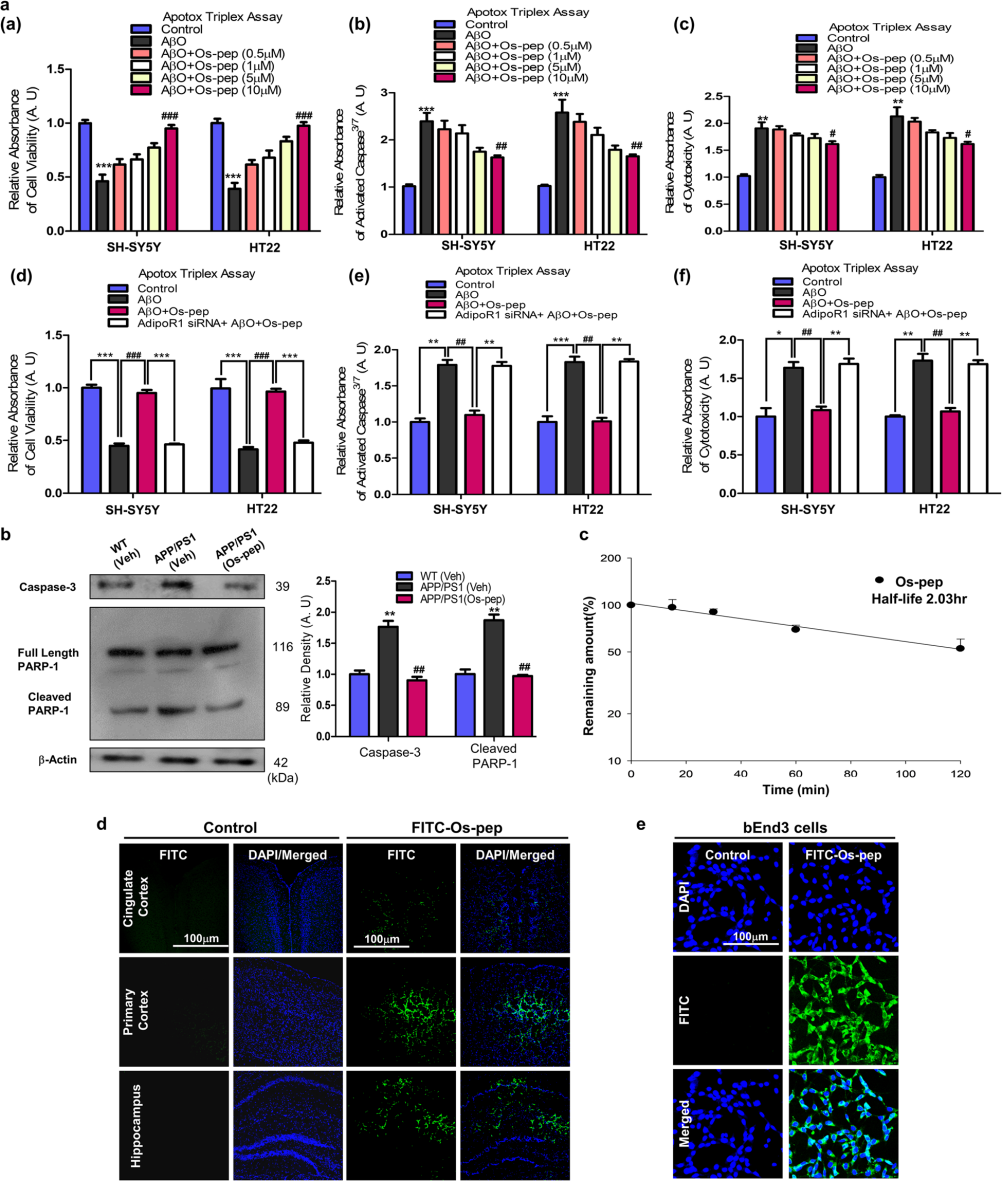
Neuroprotective and pharmacokinetic profile of Os-pep in the in vivo and in vitro studies. **(a, panels** (**a-c**)) Apotox-Glo Triplex assays of the cell viability, cytotoxicity and activated caspase^3/7^ levels in SH-SY5Y and HT22 cells treated with AβO (1 μM) and different concentrations of Os-pep. (**a, panels (d-f**)) Apotox-Glo Triplex assays of the cell viability, cytotoxicity and activated caspase^3/7^ levels in SH-SY5Y and HT22 cells transfected with the AdipoR1 siRNA and treated with AβO (1 μM) and Os-pep (10 μM). Data are expressed as means ± SEM for three independent experiments. Significance = *p < 0.05, **p < 0.01 ***p < 0.001; #*p* < 0.05, ##*p* < 0.01, ###*p* < 0.001; One-way ANOVA followed by Turkey’s post hoc test. **(b)** Immunoblotting and quantification of activated caspase-3 and cleaved PARP-1 levels in the APP/PS1 mice. Data are expressed as the means ± SEM for the indicated proteins (n = 8 mice/group), and the number of independent experiments = 3. The data presented in this figure are normalized to control values. Significance = **p < 0.01; ##*p* < 0.01; One-way ANOVA followed by Turkey’s post hoc test*.* **(c-e)** Pharmacokinetic profile of Os-pep. **(c)** Stability of Os-pep in mouse plasma. The Os-pep concentration at 0 min is set to 100%. Bars represent SD (standard deviation) (*n* = 3) for triplicate samples. **(d)** Confocal microscopy images of the Veh-injected WT and Os-pep-FITC-injected WT mice. The number of mice = 3/group and number of independent experiments = 3. Magnification: 10X. Scale bar = 100 μm. **(e)** Confocal microscopy images of the bEnd cells exposed to bidistilled water and Os-pep-FITC. The number of independent experiments = 3. Magnified 10X. Scale bar = 100 μm

Furthermore, to assess the neuroprotective profile of Os-pep *in vitro,* we performed ApoTox-Glo™ Triplex assays in SH-SY5Y and HT22 cells. The results of the ApoTox-Glo™ Triplex assay in SH-SY5Y and HT22 cells showed that cell viability was reduced, whereas cytotoxicity and the activation of caspases^3/7^ were increased after AβO (1 μM) exposure for 12 h. However, cotreatment with varying concentrations (0.5, 1, 5 and 10 μM) of Os-pep reduced the neurotoxic effects of AβO (1 μM); notably, the 10 μM concentration was more effective and significantly reduced AβO (1 μM) neurotoxicity by increasing cell viability and decreasing cytotoxicity and caspase^3/7^ activation (Fig. 8a, panels (a-c)). We then analyzed the protective effects of Os-pep (10 μM) on AβO (1 μM)-treated SH-SY5Y and HT22 cells transfected with AdipoR1 siRNA using ApoTox-Glo™ Triplex assays. The cell viability, cytotoxicity and caspases^3/7^ detection results indicated that Os-pep (10 μM) did not reduce the AβO-induced toxicity in the AdipoR1 siRNA-transfected SH-SY5Y and HT22 cells (Fig. 8a, panels (d-f)).

Next, to assess the neuroprotective effects of Os-pep *in vivo*, we evaluated the levels of apoptotic and neurodegenerative markers, e.g., activated caspase-3 and cleaved poly (ADP-ribose) polymerase-1 (PARP-1), by immunoblotting. Os-pep administration (5 μg/g, i.p., on alternating days for 45 days) significantly decreased the activated caspase-3 and cleaved PARP-1 levels in the APP/PS1 mice (Fig. 8b). Notably, and most importantly, cleaved PARP-1 was detected by stripping the PVDF membrane used for the detection of AdipoR1, so these results confirmed that AdipoR1 activation by Os-pep plays a key role in protection against neurodegenerative diseases. Further studies are required to explore this mechanism. Furthermore, based on these results, Os-pep exerted a beneficial protective effect by activating AdipoR1, which is consistent with other studies, suggesting that AdipoR1 activation prevents neurodegeneration [11–16, 38, 39].

Next, we performed pharmacokinetic analysis, and Os-pep was degraded according to first-order kinetics. After 2 h, 53% of the initial concentration remained. The Os-pep half-life (t_1/2_) was calculated to be approximately 2 h (Fig. 8c). The 2 h t_1/2_ of Os-pep indicated that Os-pep is a relatively stable peptide compared to other bioactive peptides, affirming its stability. Furthermore, we determined the ability of Os-pep to cross the blood-brain barrier (BBB) and reach the brain. Based on confocal microscopy, FITC-conjugated Os-pep reached the brain after an i.p. injection (Fig. 8d). In addition, we also analyzed the cellular uptake of Os-pep using FITC-conjugated Os-pep in brain endothelial (bEnd3) cells (Fig. 8e). Based on these *in vitro* and *in vivo* results, the small 9-amino acid Os-pep represents a safe and stable neuroprotective adiponectin-mimetic novel peptide that crosses the BBB and reaches the brain, where it stimulates AdipoR1 and prevents memory deficits in aberrant brain metabolism-associated AD.

## Discussion

According to several recent studies, the reduction of adiponectin and AdipoR1 signaling in the brain has been implicated in metabolic-associated AD. The reduction of adiponectin levels and adiponectin receptor signaling may be emerging factors that increase the risk of AD associated with metabolic dysfunction. Subjects with adiponectin and AdipoR1 signaling deficiencies developed brain insulin resistance and impaired downstream insulin signaling, which mediates AD pathology [9, 14–16, 22]. For the past decade, our group has investigated whether the phytopolypeptide osmotin (a homolog of mammalian adiponectin) reduces AD pathology. However, osmotin and adiponectin are limited as therapeutics for use in further translational and clinical studies because of their large size. Therefore, we extended our approach by using the osmotin-based small adiponectin-mimetic novel nonapeptide Os-pep. Furthermore, to the best of our knowledge, for the first time, we explored the in-depth mechanistic biological and therapeutic effects of the adiponectin-mimetic novel nonapeptide on rigorous *in vitro* and *in vivo* models of AD and Adipo^−/−^ mice. We verified the results in AβO-treated, AβO- and scramble and functional AdipoR1 shRNA-treated, and APP/PS1 mice, in *in vitro* AβO and APPswe/ind overexpression AD models, and in HFD and Adipo^−/−^ mice.

Many studies have investigated whether AβO induces brain metabolic dysfunction, which induces neuronal insulin resistance in the brains of AD patients and in* in vitro* and animal AD models; dysregulation of insulin signaling through the phosphorylation of p-IRS-1^Ser636/312^ induces synaptic and memory impairments [8, 9]. Suppression of AdipoR1/AMPK signaling in Adipo^−/−^ mice leads to the development of insulin resistance in the brain and subsequent development of AD pathology [15, 16]. Notably, aberrant neuronal insulin signaling and brain insulin resistance increase Aβ generation by enhancing amyloidogenic APP processing, and this may lead to a vicious cycle. Aβ impairs insulin signaling and, in turn, reduces p-PI3K/Akt/GSK3β^Ser9^ signaling. Activated insulin and p-PI3K/Akt/GSK3β^Ser9^ signaling have been shown to play key roles in the reduction of Aβ and tau pathology [49–51]. Deficits in AdipoR1 signaling in chronic adiponectin-deficient mouse models were recently shown to mediate AD pathology, leading to an increase in p-IRS-1^Ser 636/312/616^ and a decrease in p-IRS-1^Tyr632^ levels. These changes result in the downregulation of the p-PI3K/Akt/p-GSK3β^Ser9^ pathway, leading to GSK3β activation and the subsequent escalation of Aβ production. Furthermore, adiponectin induces AdipoR1/AMPK signaling to increase insulin sensitivity through the activation of p-IRS-1^Tyr632^ and downstream insulin signaling via stimulation of the p-PI3K/Akt/GSK3β^Ser9^ pathway [9, 11, 15, 16]. Therefore, adiponectin, adiponectin-mimetic peptides and AdipoR1 have been implicated in various metabolic diseases and are key targets for the prevention of neurological disorders. AdipoR1 is a primary target in AD, as studies from our group and other groups have recently reported the reduced expression of AdipoR1 in APP/PS1 and Adipo^−/−^ mice. Most importantly, we also found reduced AdipoR1 expression in the aged human AD brain. Thus, AdipoR1 activation reduces AD neuropathology and has been considered an emerging therapeutic approach for the treatment of AD [14–16, 21, 38–40]. AMPK, a key energy sensor and well-known downstream kinase of AdipoR1, is activated both *in vivo* and *in vitro* by adiponectin and other agents that mimic adiponectin via AdipoR1. AMPK exerts a neuroprotective effect against various neurodegenerative injury, and reduced AMPK expression leads to neurodegeneration. In contrast, AMPK activation regulates neurodegeneration [35, 36, 52–54]. Activated AMPK regulates aberrant brain metabolism and reduces insulin resistance [14–16, 55, 56]. Furthermore, these results are supported by those of a recent study showing that reduced AdipoR1/AMPK signaling, and neuronal insulin resistance are associated with AD pathology in adiponectin-deficient mice. According to the results from a few recent and exciting studies, activation of AdipoR1/AMPK signaling in the brain may be a beneficial therapeutic approach for the reduction of AD pathology [15, 56, 57]. The *in vitro* AβO-exposed and APPswe/ind-overexpressing cells and the *in vivo* AD and Adipo^−/−^ mice showed reduced levels of AdipoR1/p-APMK, suggesting that brain metabolism and energy are implicated in neuronal insulin resistance associated with adiponectin deficiency. Intriguingly, Os-pep, as an adiponectin-mimetic nonapeptide, had beneficial effects by activating AdipoR1/AMPK signaling to prevent neuronal insulin resistance by reducing p-IRS-1^Ser636/312^ levels and stimulating the p-IRS-1^Tyr632^ and p-PI3K/Akt/GSK3β^Ser9^ pathways through an AdipoR1/AMPK-dependent mechanism.

Synaptic dysfunction is a complex process that occurs either directly through AβO or indirectly through other intermediate parameters that contribute to synaptic deficits [58, 59]. Based on accumulating evidence, deficits in pre- and postsynaptic protein markers are implicated in memory decline in AD. Importantly, AβO is known to suppress LTP and induce memory dysfunction [59–61]. In brain metabolism, AD reduces synaptic density by impairing and inhibiting insulin signaling in the brain. However, activation of insulin signaling and reduction of neuronal insulin resistance in the brain prevents synaptic/memory deficits in AD [62, 63]. Recently, brain neuronal insulin resistance was reported to trigger synaptic plasticity dysfunction and reduce LTP and memory function in HFD mice [64]. AdipoR1 activation was recently shown to increase the levels of pre- and postsynaptic proteins and synaptic protection in HFD mice in *in vivo* and *in vitro* AD models as well as in Adipo^−/−^ mice [14–16]. Furthermore, our recent studies indicated that activated AdipoR1 regulated neuronal outgrowth and synaptic complexity in AD [40]. Most importantly, in this study, Os-pep, as an adiponectin-mimetic nonapeptide that functions as a ligand for AdipoR1 to trigger AdipoR1/AMPK signaling, subsequently decreased neuronal insulin resistance and increased pre- and postsynaptic protein markers as well spine morphogenesis in the hippocampal neuronal CA1 region of Adipo^−/−^ and AβO mice. Moreover, we observed for the first time that the dosage regimen of Os-pep improved LTP and cognitive functions such as learning and memory via the activation of AdipoR1 in normal AβO-injected and transgenic APP/PS1 mouse models of AD and in Adipo^−/−^ mice, as measured using the MWM and Y-maze tests.

Adiponectin and AdipoR signaling were recently shown to play a promising role in various metabolic and age-associated neuropathological disorders because adiponectin enhances peripheral and central insulin sensitivity and prevents insulin resistance [65–68]. Importantly, AdipoR1 has been implicated in the regulation of AD-related neuropathology*.* Adiponectin activates AdipoR1 and prevents neuroinflammation and shows protective activities in various metabolic disorders. Hence, adipokines and their receptors, particularly adiponectin and its mimetic peptides via AdipoR1 activation, represent an emerging, novel and attractive potential therapeutic target for the prevention of AD and other neuropathological disorders [14–16, 21, 35–37, 69–73]. However, adipokines, particularly adiponectin and its receptor, should be thoroughly monitored for translation-based studies because of recent findings and epidemiological studies that reported that an excess adiponectin level is associated with lethal effects, such as circulatory disorders (chronic heart failure & chronic kidney failure), as well as other undesirable effects, such as weight loss, low skeletal muscle mass/density, and physical functioning impairment, which ultimately lead to morbidity and mortality [26–31].

## Conclusion

In summary, our intriguing *in vitro* and *in vivo* findings strongly indicated that an adiponectin-mimetic novel nonapeptide crossed the BBB and had a good safety and protection profile, regulated neuronal metabolism-associated AdipoR1/AMPK targets, prevented neuronal insulin resistance, enhanced downstream insulin signaling, and consequently improved synaptic plasticity and memory functions in AD and Adipo^−/−^ mice. Previously studies demonstrated that osmotin derived this Os-pep [43, 44], interacts with the mammalian adiponectin recognition site on AdipoR1 as well as in our pilot studies and the finding in this study demonstrated that Os-pep induced beneficial and therapeutic effects via interacting primarily with AdipoR1, which subsequently activate AdipoR1 and its downstream signaling. However, it is worth to mention here that the Os-pep might has the potential to activate AdipoR2 or activate or antagonize other non-specific receptors and transcription factors, which will be further investigated in computational approaches as well as *in vitro* and *in vivo* models of other peripheral-and brain metabolic disorders, and other neurodegenerative diseases. Recently, the therapeutic approach of using peptides to prevent neurological disorders has emerged, and its advantages over the use of small molecules have been reported because peptides display higher potency, specificity and fewer side effects in other targets in the body. In addition, peptide therapy is economical, nontoxic and safe. All of these parameters and the increased accessibility via easy customization and synthesis in a well-controlled system make the use of peptides an ideal and rational therapeutic strategy for the development of drugs against AD. Based on our rigorous results, we believe and suggest that Os-pep is an emerging, interesting and valuable novel peptide-based therapeutic candidate for translational research to halt and treat AD. Of note, considering the adiponectin paradox and the very recent amyloidogenic evolvability theory [24, 25, 74], we suggest that future translational studies of this adiponectin-mimetic nonapeptide be performed, and the therapeutic effects of Os-pep on Aβ aggregation and the hyperphosphorylation of tau proteins as well as the regulation of cellular homeostasis will be tested in animal models at different stages of AD, such as transgenic mice that are 12 ~ 24 months old as well as SAMP8 (senescence-accelerated) mice as late-onset (sporadic) AD models. Similarly, we recommend that Os-pep-based therapeutic approaches be validated for the treatment of other neurodegenerative disorders associated with aberrant metabolism.

## Supplementary Information

**Additional file 1 **Supplementary **Table 1. Fig. S1** Os-pep stimulated AdipoR1/p-AMPK signaling in vitro AD models. **Fig. S2** Os-pep attenuated neuronal insulin resistance in HFD mice. **Fig. S3** Os-pep regulated various plasma serum biochemical parameters and the body weight of the APP/PS1 and HFD mice. **Fig. S4** Os-pep regulated various plasma serum biochemical parameters and the body weight Adipo−/− mice. **Fig. S5** Os-pep regulated dendritic complexity and spine density in the AβO-treated mice. **Fig. S6** Path length and swimming speed of the AβO-treated, APP/PS1 and Adipo−/− mouse models during the MWZ test.

## Data Availability

All data required to validate our hypotheses in the paper are provide in the original manuscript and supplementary material file. Further any additional data related to this manuscript will be available upon request from the corresponding author.

## Abbreviations

AdipoR1: Adiponectin receptor 1
AD: Alzheimer’s disease
Adipo−/−: Adiponectin knockout
AβO: Amyloid beta oligomer
ALS: Amyotrophic lateral sclerosis
AMPK: AMP-activated protein kinase
ANOVA: Analysis of variance
APP/PS1: B6.Cg-Tg(APPswe,PSEN1dE9)85Dbo/Mmjax (Amyloid precursor protein and presenilin 1)
BBB: Blood-brain barrier
bEnd3: Brain endothelial cell line
Comp. C: Compound. C
CLSM: Confocal laser scanning microscopy
CSF: Cerebrospinal fluid
DAPI: 4′, 6′-Diamidino-2-phenylindole
DMEM: Dulbecco’s Modified Eagle’s Medium
DMSO: Dimethyl sulfoxide
ELISA: Enzyme-linked immunosorbent assay
FBS: Fetal bovine serum
FFA: Free fatty acids
fEPSPs: Field excitatory postsynaptic potentials
FITC: Fluorescein isothiocyanate
HDL: High density lipoprotein
HFD: High fat diet
I.C.V: Intracerebroventricular
IP: Intraperitoneal
LC-MS/MS: Liquid chromatography-tandem mass spectrometry
LDL: Low density lipoprotein
LTP: Long term potentiation
MWM: Morris water maze
PBS: Phosphate buffered saline
PD: Parkinson’s disease
PEI: Polyethylenimine
PARP-1: Poly (ADP-ribose) polymerase-1
PSD-95: Post-synaptic density protein
siRNA: Small interfering RNA
Synap: Synaptophysin
TC: Total cholesterol
TG: Triglycerides
TIF: tagged image file format
TRITC: Tetramethyl rhodamine isothiocyanate
Veh: Vehicle
WT: Wild-type
