# Supplementary material for: Adiponectin-mimetic novel nonapeptide rescues aberrant neuronal metabolic-associated memory deficits in Alzheimer’s disease
Source: Mol Neurodegener. 2021 Apr 13;16:23. doi: 10.1186/s13024-021-00445-4 (PMC8042910; doi:10.1186/s13024-021-00445-4)
Supplement: Supplementary file 1 — Additional file 1 Supplementary Table 1. Fig. S1 Os-pep stimulated AdipoR1/p-AMPK signaling in vitro AD models. Fig. S2 Os-pep attenuated neuronal insulin resistance in HFD mice. Fig. S3 Os-pep regulated various plasma serum biochemical parameters and the body weight of the APP/PS1 and HFD mice. Fig. S4 Os-pep regulated various plasma serum biochemical parameters and the body weight Adipo−/− mice. Fig. S5 Os-pep regulated dendritic complexity and spine density in the AβO-treated mice. Fig. S6 Path length and swimming speed of the AβO-treated, APP/PS1 and Adipo−/− mouse models during the MWZ test. [file 13024_2021_445_MOESM1_ESM.docx]

**Revised Supplementary Material (MOND-D-20-00214R5)**

**Adiponectin-mimetic** **novel nonapeptide rescues aberrant neuronal metabolic-associated memory deficits in Alzheimer’s disease**

Tahir Ali^1^, Shafiq Ur Rehman^1^, Amjad Khan^1^, Haroon Badshah^1^, Noman Bin Abid^1^, Min Woo Kim^1^, Myeung Hoon Jo^1^, Seung Soo Chung^2^, Hyoung-gon Lee^3^, Bart P.F. Rutten^4^, and Myeong Ok Kim^1^*

Running Title: Novel nonapeptide rescues memory deficits

^1^Division of Applied Life Science (BK 21), College of Natural Science, Gyeongsang National University, Jinju, 52828, Republic of Korea. tahirneuro@gmail.com (T. A); [shafiq.qau.edu@gmail.com](mailto:shafiq.qau.edu@gmail.com) (S.U.R); amjadkhan@gnu.ac.kr (A.K); [haroonbacha87@yahoo.com](mailto:haroonbacha87@yahoo.com) (H.B); [noman_abid@gnu.ac.kr](mailto:noman_abid@gnu.ac.kr) (N.B.A); [mwkim0322@gnu.ac.kr](mailto:mwkim0322@gnu.ac.kr) (M.W.K); [audgns1217@gnu.ac.kr](mailto:audgns1217@gnu.ac.kr) (M.H.J); [mokim@gnu.ac.kr](mailto:mokim@gsnu.ac.kr) (M.O.K).

^2^Department of Physiology, College of Medicine, Yonsei University, Seoul 120-752, Republic of Korea. [SSCHUNG@yuhs.ac](mailto:SSCHUNG@yuhs.ac) (S.S.C).

^3^Department of Biology, The University of Texas at San Antonio, USA. [hyoung-gon.lee@utsa.edu](mailto:hyoung-gon.lee@utsa.edu) (H.L).

^4^Translational Neuroscience and Psychiatry, School for Mental Health and Neuroscience (MHeNs), Maastricht University Medical Centre, Maastricht, Netherlands. [b.rutten@maastrichtuniversity.nl](mailto:b.rutten@maastrichtuniversity.nl) (B.P.F.R).

*Corresponding author

Myeong Ok Kim, Prof. Ph.D.

Head of the Brain-Metabolic Neurodegenerative Disease Center

Division of Applied Life Science, College of Natural Sciences,

Gyeongsang National University, Jinju, 52828, South Korea

Tel.: +82-55-772-1345, Fax: +82-55-772-2656, E-mail: mokim@gnu.ac.kr

**Materials & methods**

**Materials**

The dimethyl sulfoxide (DMSO), SPD304 and insulin were bought from Sigma Chemical Co. (St. Louis, MO, USA). The Bio-Rad protein assay kit was purchased Bio-Rad Laboratories, CA, USA. The 4-12% Bolt^TM^ Mini Gels, 1x MES SDS running buffer, and iBolt Gel Transfer Stacks PVDF, regular and mini were obtained from Novex, Life Technologies, Kiryat Shmona, Israel. The Immunobilon-P-transfer membrane was purchased from EMD Millipore (Billerica, MA). Dulbecco’s Modified Eagle’s Medium (DMEM) was obtained from Gibco Life Sciences. The SH-SY5Y (Human neuroblastoma) cells were bought from the Korean Cell line Bank, KCLBNO (22266). The HT22 (mouse hippocampal) cells were provided by Prof. Koh (Gyeongsang National University, Republic of Korea). Chamber slides and 96-well plates were obtained from Thermo Fisher Scientific (75 Panorama Creek Drive Rochester, NY14625-2385, USA). The ApoTox-Glo^TM^ Triplex assay kits were purchased from Promega (Madison, WI).

**Development of the HFD mouse model**

A separate cohort of animals was used to develop the HFD mouse model. Eight-week-old C57BL/6N male mice were bought from Samtako Bio (Republic of Korea). Mice were housed in the university animal housing facility under a 12 h/12 h light/dark cycle at 23°C and 60 ± 10% humidity, with food and water available ad libitum. After one week of acclimation, the mice have divided into the following groups: control mice fed normal chow, mice in the HFD groups fed an HFD (protein: 20%, carbohydrate: 20% and fat: 60%) for 2 months. The HFD mice were further divided into two additional groups. Control mice (WT, Veh) were fed normal chow and HFD mice were either treated with Veh (HFD-Veh) on alternating days for 45 days and or with Os-pep (5 μg/g, i.p., on alternating days for 45 days) (HFD-Os-pep).

***In vitro* induction of neuronal insulin resistance**

Human neuroblastoma SH-SY5Y cells were treated with 1 μM of insulin for 48 h to produce intrinsic insulin-resistant human neuroblastoma (SH-SY5YIR) cells. After 48 h, SH-SY5YIR cells were overexpressed with human APPswe/ind and subjected with AdipoR1 siRNA which further exposed to 10 nM insulin, Os-pep (10 μM) or compound C (10 μM) for 12 h, and then cell lysates were collected and proceeded accordingly for immunoblotting and ELISA.

***In vitro* culture of SH-SY5Y cells for ELISA and AMPK assays**

SH-SY5Y (2×10^4^/ml) cells used for ELISA and AMPK assays were cultured in 35-mm dishes in the presence of culture medium of DMEM containing FBS (10%) and antibiotics (1%) at 37°C in humidified air containing 5% CO_2_ to 70-75% confluences. Cells were overexpressed with the pCAX vector containing the human APPswe/ind gene using Lipofectamine 3000 (Life Technologies), according to the manufacturer’s instructions. The AdipoR1 siRNA (h) (SC-60123) and AMPK siRNA (h) (SC-45312) (Santa Cruz Biotechnology, Inc) were used at a concentration of 10 μM per transfection and allowed to express for 72 h, according to the manufacturer’s protocol (Santa Cruz Biotechnology, Inc.). The negative siRNA (Ambion, Thermo Fisher Scientific) was used as a control. Seventy-two hours after transfections, cells were treated with Os-pep (10 μM) for 12 h and cell lysates were collected and analyzed using ELISAs and immunoassays, as appropriate.

**AdipoR1 ELISAs**

The AdipoR1 expressions were measured in the brain homogenates from APP/PS1 and Adipo^−/−^ mice and in lysates from APPswe/ind-overexpressed SH-SY5Y cells transfected with the AdipoR1 siRNA and treated Os-pep (10 µM) *in vitro* using the mouse and human AdipoR1 ELISA kits, accordingly to the provider’s instructions (MyBioSource).

**AMPK kinase assays**

The CycLex® AMPK kinase assay kit was used to analyze the levels of activated AMPK in brain homogenates from APP/PS1 and Adipo^−/−^ mice and in lysates from APPswe/ind-overexpressed SH-SY5Y cells expressed with the AdipoR1 siRNA and exposed to Os-pep (10 µM) *in vitro*, accordingly to the provider’s instructions (MBL International Corporation).

**Biochemical analyses of plasma serum and body weight measurements of the mice**

We monitored and weighed the mice three times per week to examine the effect of Os-pep dosage regimen (5 μg/g, i.p., on alternating days for 45 days) on the animals’ body weights. At the completion of the treatment, we calculated the average change in the body weight of the mice. At the completion of the treatments and behavioral experiments, blood samples were collected by cardiac puncture of the right atrium. The serum was collected from whole blood by centrifugation (3000 rpm for 10 min) at room temperature and was stored at -20 °C until it was processed for biochemical analyses by SCL Labs (Seoul Medical Science Institute) to evaluate various metabolic parameters, e.g., glucose (Glu), free fatty acids (FFA), total cholesterol (TC), triglycerides (TG), high density lipoprotein (HDL) and low density lipoprotein (LDL) levels.

**Preparation of mouse brain sections for morphological analyses**

After behavioral analyses, the animal (number of mice = 5/per group) transcardially were perfused using ice-cold paraformaldehyde (4%), and the brain brains were post-fixed in 4% paraformaldehyde for 72 h and transferred to 20% sucrose for 72 h. Brains were frozen in O.C.T. compound (A.O, USA), and 12-14 μm coronal sections were cut using a CM 3050C cryostat (Leica, Germany). Sections were thaw-mounted on ProbeOn Plus charged slides (Fisher, USA).

**The stability and half-life of Os-pep in mouse plasma**

The stability of Os-pep in mouse plasma was evaluated. An aqueous stock solution of Os-pep was added to each mouse plasma sample (n= 3) at a final concentration of 1 μg/mL. Tubes were placed in a Thermomixer (Eppendorf, Germany) and maintained at 37 °C with rotation at 300 rpm. These samples were incubated for up to 2 h. The amount of peptide remaining after the incubation was immediately determined by liquid chromatography-tandem mass spectrometry (LC-MS/MS). At different time intervals, an aliquot (100 μL) of ice-cold acetonitrile containing 0.1 μg/mL gabapentin (internal standard) was added to a 50 μL aliquot of the sample. Deproteinized samples were analyzed after vortex-mixing and centrifugation.

**The bEnd3 cell culture and assessment of Os-pep uptake using confocal microscopy**

The uptake of Os-pep-FITC by mouse bEnd3 cells (ATCC, USA), which served as an *in vitro* BBB model, was analyzed using confocal laser scanning microscopy (CLSM). The bEnd3 cells (2×10^4^/ml) were cultured in chamber slides (Thermo Fisher Scientific 75 Panorama Creek Drive Rochester, NY14625-2385, USA) in DMEM supplemented with 10% FBS and 1% antibiotics at 37°C in humidified air containing 5% CO_2_. After the cells reached 70-80% confluence, they were exposed to FITC-conjugated Os-pep (10 μM) for 12 h; cells in the control group were exposed to bidistilled water in the culture medium under the same conditions. Afterward, cells were washed with 0.01 M PBS, fixed with 4% paraformaldehyde, and again washed with 0.01 M PBS. Slides were mounted with DAPI and Prolong Antifade Reagent (Molecular Probe, Eugene, OR, USA). Representative images were captured using FITC and DAPI filters with a laser confocal FluoView FV 1000 microscope equipped with FV10-ASW 3.1 Viewer (Olympus, Tokyo, Japan).

**Assessment of Os-pep uptake *in vivo* using confocal microscopy**

A separate cohort study was designed using male C57BL/6N WT mice. FITC-conjugated Os-pep (5 μg/g) and the same volume of saline was i.p. injected into the WT mice (n=3 mice/group). One hour after the injection, the mice were transcardially perfused with 4% ice-cold paraformaldehyde, and the brains were post-fixed with 4% paraformaldehyde for 72 h and transferred to 20% sucrose for 72 h. Brains were frozen in O.C.T. compound (A.O, USA), and 14-μm coronal sections were cut using a CM 3050C cryostat (Leica, Germany). Sections were thaw-mounted on ProbeOn Plus charged slides (Fisher, USA). Slides containing tissues from the saline- and Os-pep-FITC-injected WT mice were washed twice with 0.01 M PBS for 10 min. Slides were mounted with DAPI and Prolong Antifade Reagent (Molecular Probe, Eugene, OR, USA). Images of immunofluorescence staining were captured with FITC and DAPI filters using a laser confocal FluoView FV 1000 microscope equipped with FV10-ASW 3.1 Viewer (Olympus, Tokyo, Japan).

***In vitro* protective profile of Os-pep via ApoTox-Glo^TM^ Triplex assay**

The ApoTox-Glo^TM^ Triplex assay (Promega, Madison, WI) was performed to assess the viability, cytotoxicity and caspase^3/7^ activation in the human neuroblastoma SH-SY5Y and mouse hippocampal HT22 cells. SH-SY5Y and HT22 cells (2×10^4^/ml) were cultured in 96-well plates (Thermo Fisher Scientific) containing DMEM supplemented with 10% FBS and 1% antibiotics and incubated at 37°C in a humidified incubator containing 5% CO2. After reaching 70% confluency, the cells were treated with AβO (1 μM) and different concentrations of Os-pep (0.5, 1, 5 and 10 μM) for 12 h. The control group (cells treated with 0.01% DMSO) was also analyzed. In addition, SH-SY5Y and HT22 cells were transfected with the AdipoR1 siRNA (h) (SC-60123) and AdipoR1 siRNA (m) (SC-60124), respectively, and treated with AβO (1 μM) and Os-pep (10 μM) for 12 h; the control group was transfected with the negative siRNA (Ambion, Thermo Fisher Scientific) and treated with 0.01% DMSO. The assay was performed according to our previously described protocols [38, 39].

**Supplementary table 1: Antibodies information**

| **Antibody** | **Host** | **Application** | **Manufacturer** | **Catalog**  **Number** | **Concentration** |
| --- | --- | --- | --- | --- | --- |
| AdipoR1 | Rabbit | WB/IF | abcam discover more, USA | ab-126611 | 1:1000/1:100 |
| p-IRS-1 Ser 312 | Rabbit | WB | = | ab: 66154 | 1:1000 |
| p-IRS-1 Ser 636 | Rabbit | WB/IF | = | SC-33957 | 1:1000/1:100 |
| p-IRS-1 Tyr 632 | Goat | WB | Santa Cruz  Biotechnology, USA | SC-17196 | 1:1000 |
| IRS-1 | Rabbit | WB | = | SC-559 | 1:1000 |
| p-GSK3β (Ser 9) | Mouse | WB | = | SC-37480 | 1:1000 |
| GSK3β | Rabbit | WB | = | SC-9166 | 1:1000 |
| p-PI3K | Rabbit | WB | = | SC-2931 | 1:1000 |
| PI3K | Rabbit |  |  | SC-376641 | 1:1000 |
| PARP-1 | Mouse | WB | = | SC-8007 | 1:1000 |
| Caspase-3 | Mouse | WB | = | SC: 7272 | 1:1000 |
| PSD-95 | Mouse | WB | = | SC:71933 | 1:1000 |
| Synap | Rabbit | WB | = | SC-9116 | 1:1000 |
| SNAP-23 | Mouse | WB | = | SC-374215 | 1:100 |
| β-Actin | Mouse | WB | = | SC-47778 | 1:2000 |
| p-AMPK α  (Thr 172) | Rabbit | WB | Cell Signaling, USA | 40H9S | 1:1000 |
| AMPKα | Rabbit | WB | = | 2303S | 1:1000 |
| p-Akt (Ser473) | Rabbit | WB | = | 9217S | 1:1000 |
| Akt | Rabbit | WB | = | 9272S | 1:1000 |
| SAP102 | Rabbit | WB | = | 3733S | 1:1000 |

WB: Western Blotting, IF: Immunofluorescence

**Supplementary** **figures and figure legends**

**
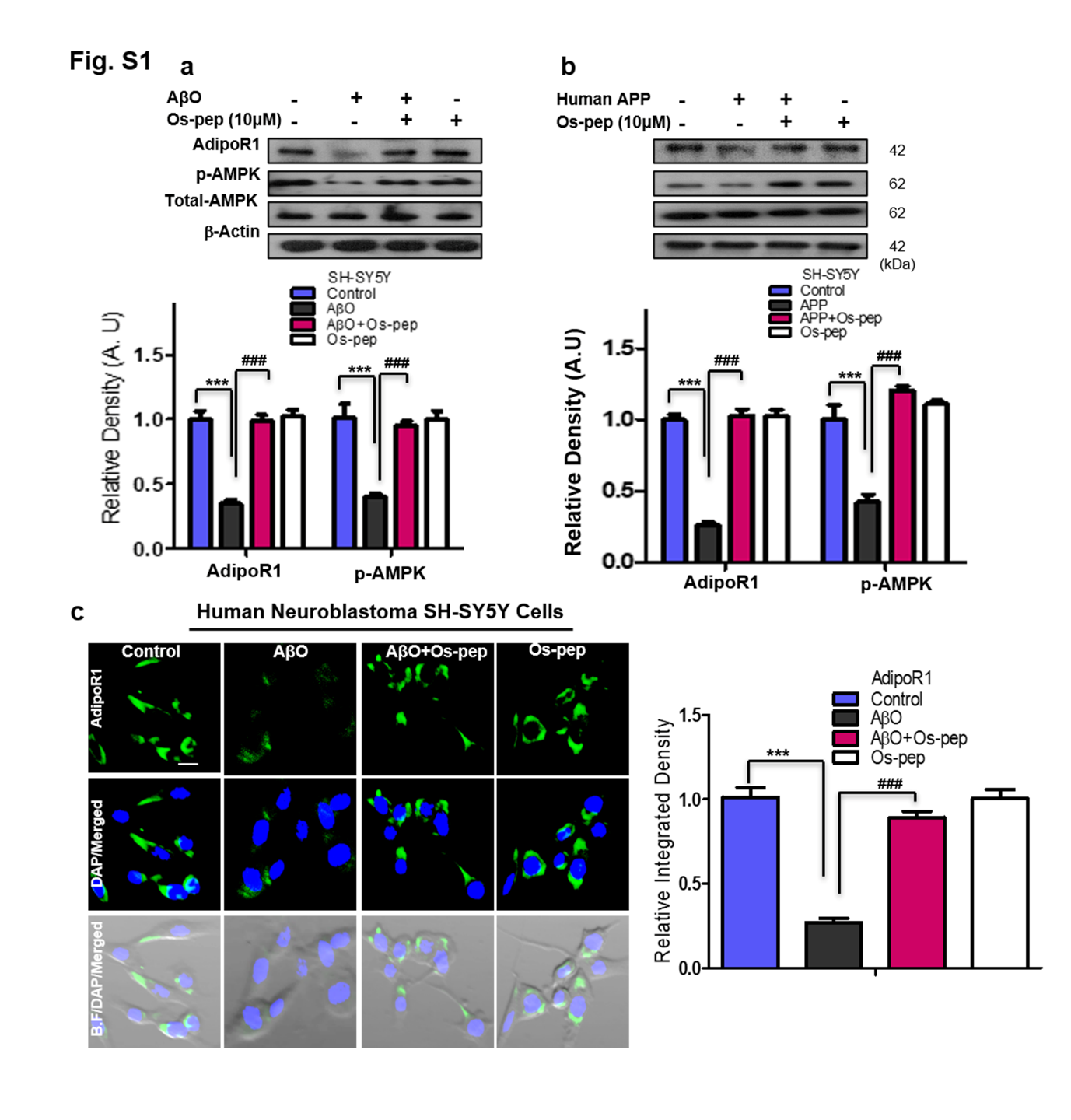
**

**Fig. S1. Os-pep stimulated AdipoR1/p-AMPK signaling *in vitro* AD models. (a, b)** Immunoblotting and quantification of AdipoR1 and p-AMPK/total-AMPK levels in the SH-SY5Y cells exposed to AβO (1 μM) or overexpressed with APPswe/ind and treated with Os-pep (10 μM) for 12 h. The data are expressed as the means ± SEM for the indicated proteins *in vitro* (n= 5/group), and the number of independent experiments= 3. Significance= ***p<0.001; ###p<0.001; One-way ANOVA followed by Turkey’s post hoc test.  **(c)** Immunofluorescence staining for AdipoR1 (green: FITC and blue: DAPI, bright field; B.F.) expression in the SH-SY5Y cells treated with AβO and Os-pep (10 μM). Magnified 40X. Scale bar= 20 μm. The data are expressed as the means ± SEM for the indicated *images* in *in vitro* (n= 5/group), and the number of independent experiments= 3. Significance= ***p<0.001; ###p<0.001; One-way ANOVA followed by Turkey’s post hoc test.

**
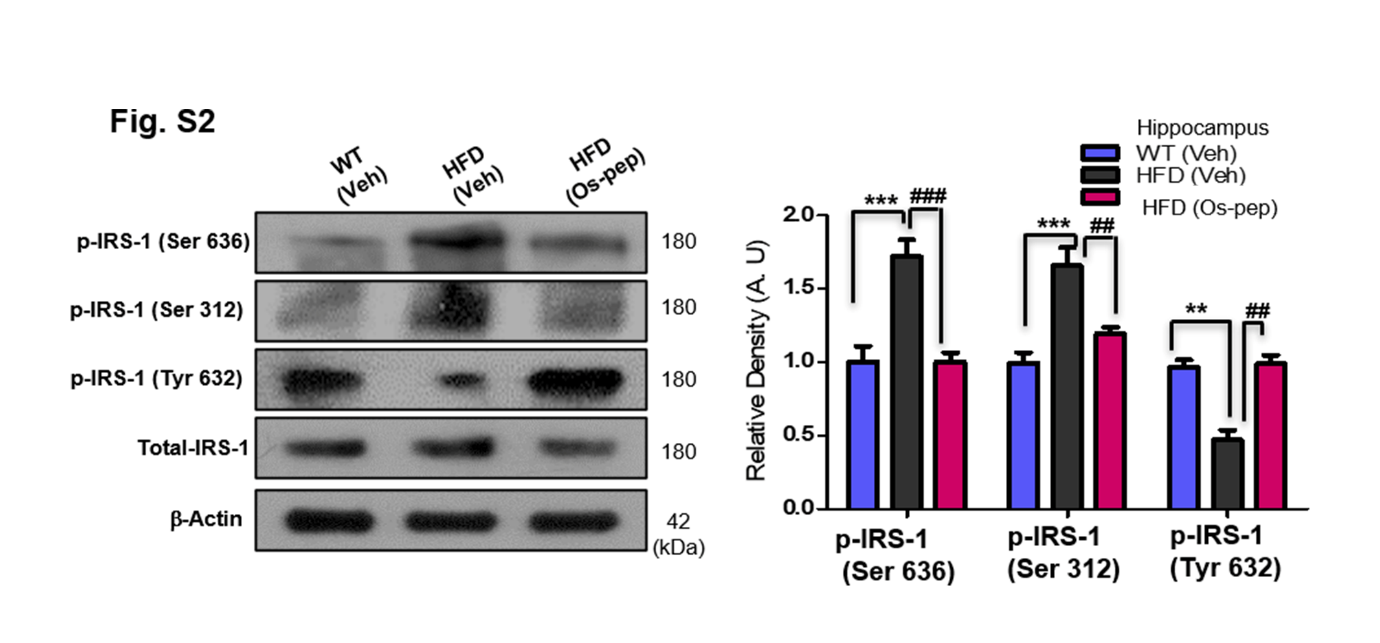
**

**Fig. S2. Os-pep rescued neuronal insulin resistance in the HFD mice. (a)** Immunoblotting and quantification of p-IRS-1 (Ser 636)/ total-IRS-1 and p-IRS-1 (Ser 312)/total-IRS-1 levels in the HFD mice. The data are expressed as the means ± SEM for the indicated proteins in vivo (n= 8 mice/group) and the number of independent experiments= 3. Significance= **p<0.01; ***p<0.001; ##p<0.01; ###p<0.001; One-way ANOVA followed by Turkey’s post hoc test.

*
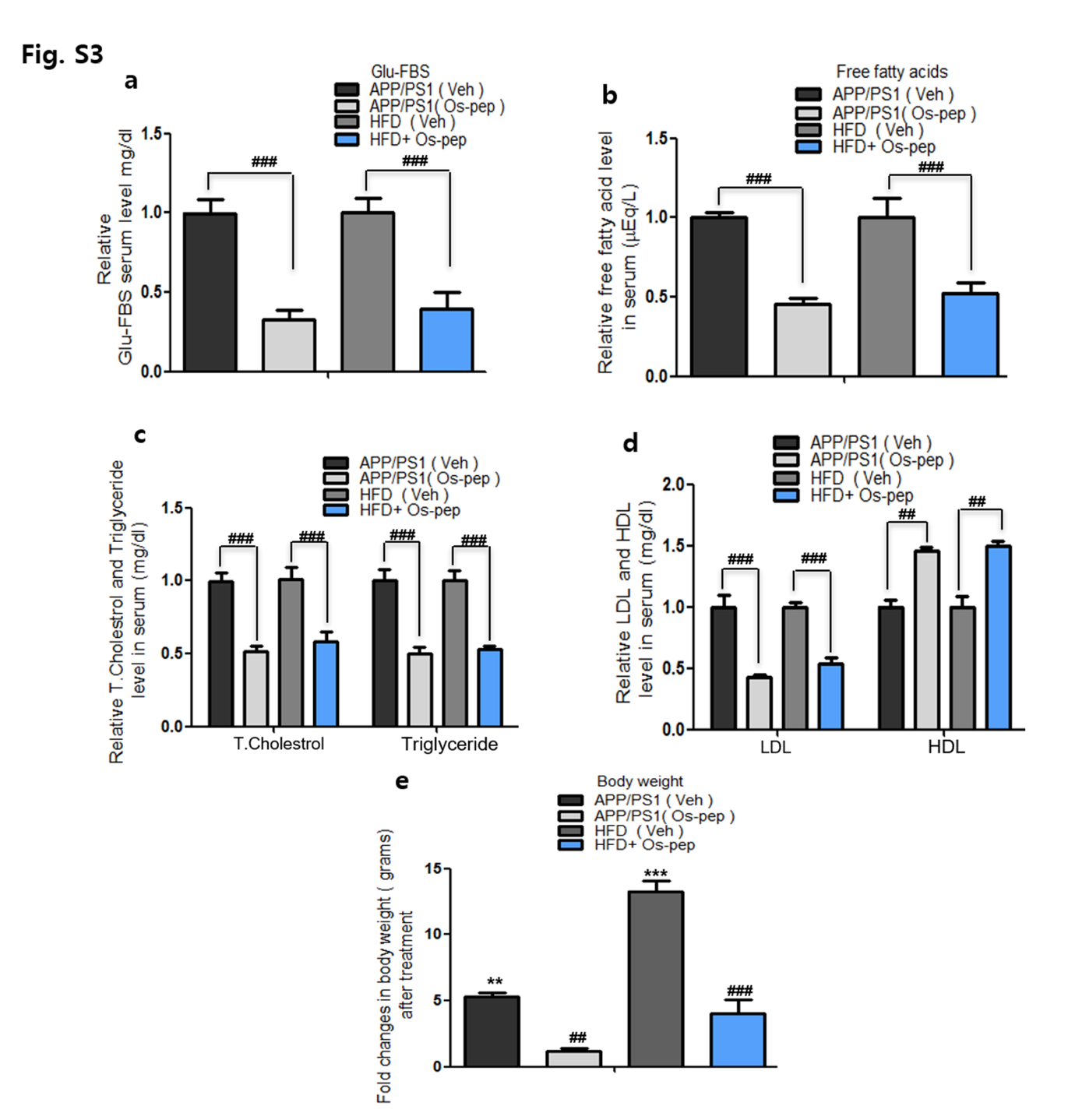
*

**Fig. S3. Os-pep regulated various plasma serum biochemical parameters and the body weight of the APP/PS1 and HFD mice.** **(a-d)** Representative histograms of the Glu-FBS, free fatty acids, total cholesterol, triglycerides, LDL and HDL levels in the serum of Veh-injected APP/PS1 and HFD mice, as well as Os-pep-treated APP/PS1 and HFD mice. The number of independent experiments= 3. **(e)** Histograms present the fold change in body weights of the APP/PS1 and HFD mice after Os-pep treatment in grams. Graphs show the means ± SEM for the mice (n= 13/group). Significance= ##p<0.01; ###p<0.001; student’s t test.

*
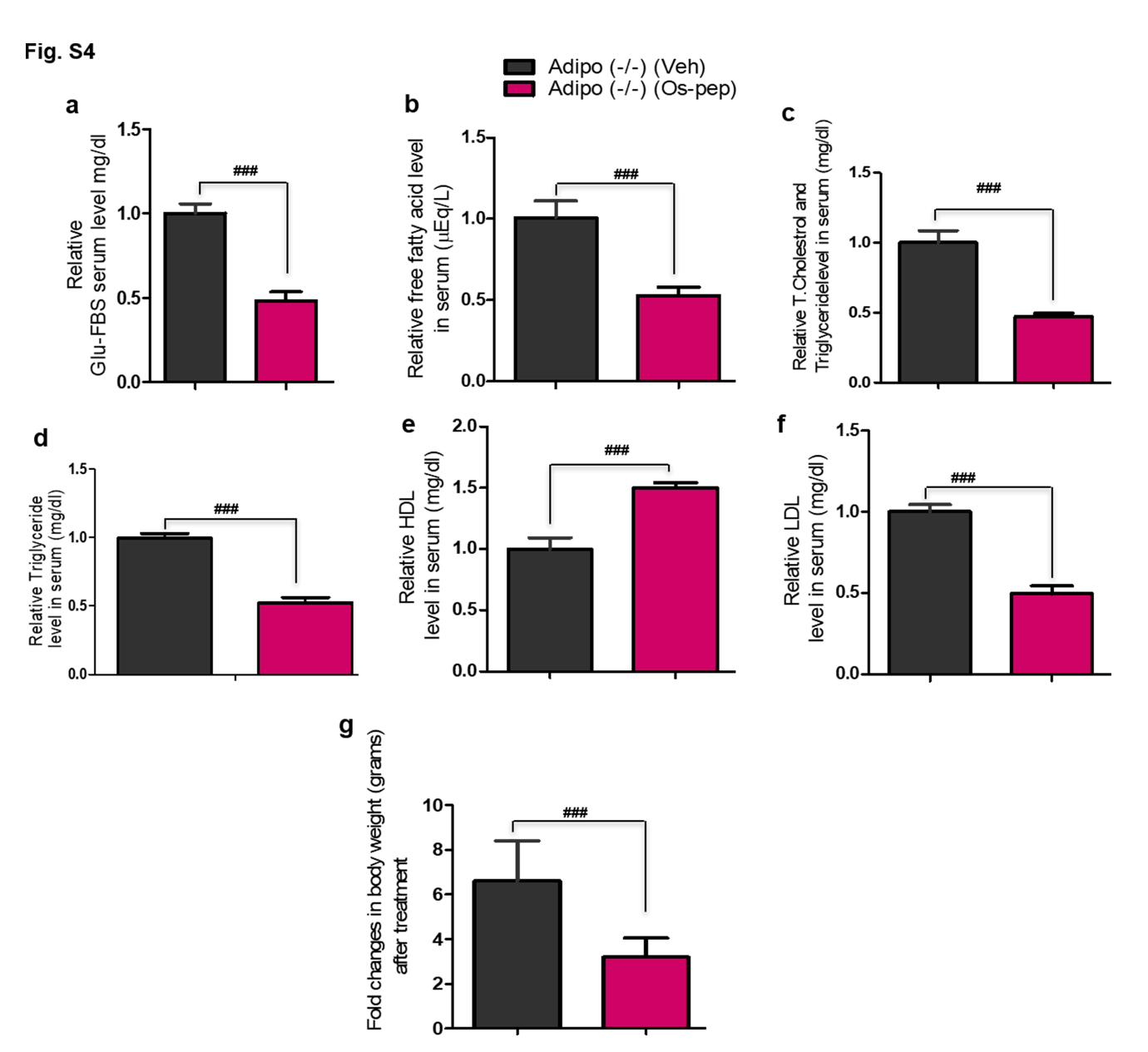
***Fig S4. Os-pep regulated various plasma serum biochemical parameters and the body weight of the Adipo^−/−^ mice. (a-d)** Representative histograms of the Glu-FBS, free fatty acids, total cholesterol, triglycerides, LDL and HDL levels in the serum of Veh-injected Adipo^−/−^ mice, as well as Os-pep-treated Adipo^−/−^ mice. The number of independent experiments= 3 (**e)** Histograms present the fold change in body weights of the Adipo^−/−^ mice after Os-pep treatment. Graphs show the means ± SEM for the mice (n= 13/group). Significance= ###p<0.001; student’s t test.

**
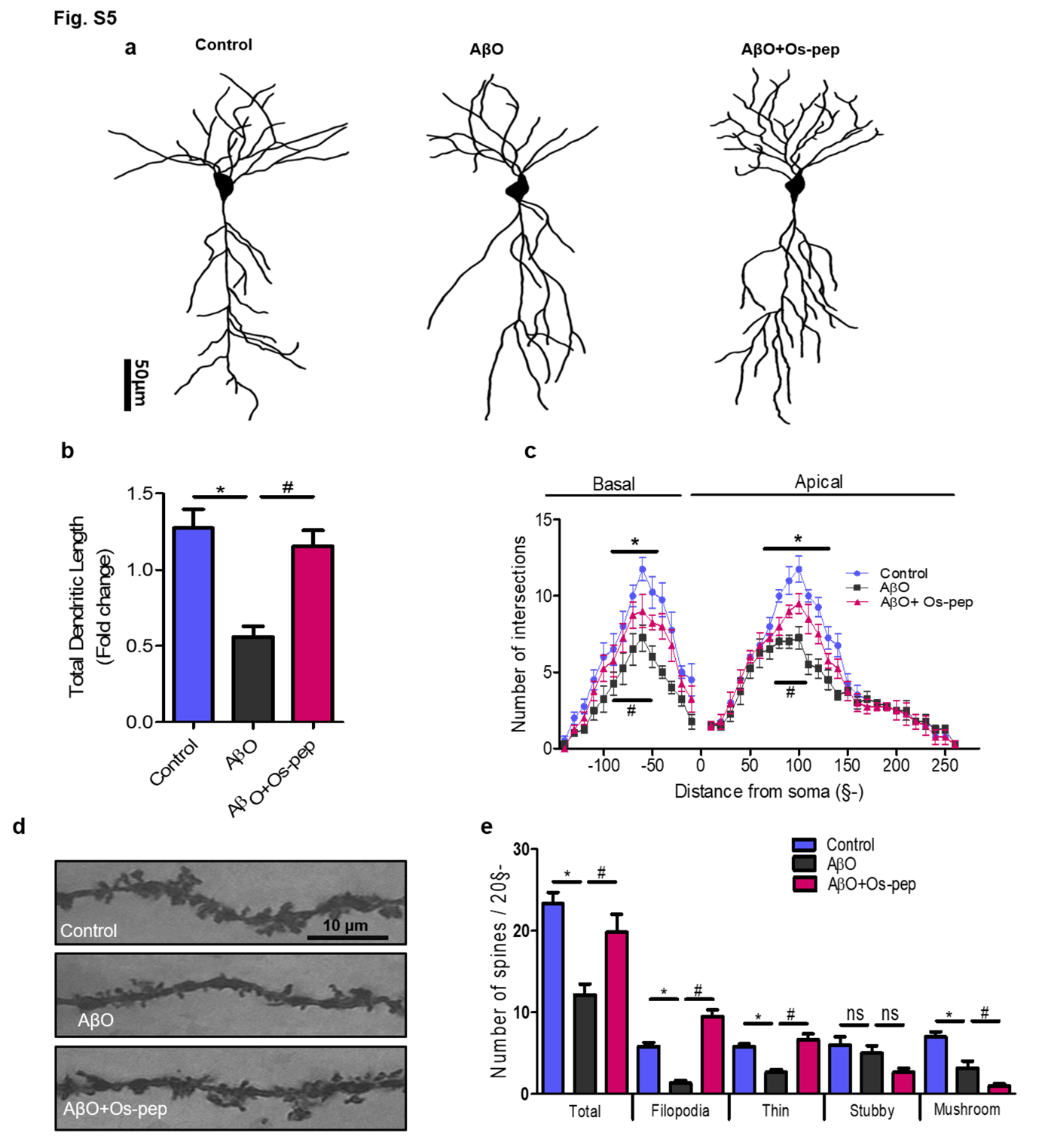
**

**Fig. S5.** **Os-pep regulated dendritic complexity and spine density in the AβO-treated mice**. **(a)** Indicated images were the example of the reconstructed hippocampal CA1 region of pyramidal neurons in the WT (Veh) , AβO and AβO (Os-pep). (**b**) Representative histogram indicated total dendritic length (sum of basal and apical dendrite length) in the hippocampal CA1 region of the mice. (**c**) Representative histogram indicated dendritic complexity via using the sholl analysis of reconstructed pyramidal neurons. (**d**) Representative images of digitalized hippocampal CA1 pyramidal dendrites from the secondary branches. (**e**) Representative histograms indicate the dendritic spines density; including the total number of spines, the number of filopodia-like, thin, mushroom and stubby spines in the hippocampal CA1 region of brain mice. The data are shown as the mean ± SEM of 45 pyramidal neurons and 300 dendritic segments per groups and the number of independent experiments= 3. Significance= *p<0.06; #p<0.05; NS=non-significant; One-way ANOVA followed by Turkey’s post hoc test.

**
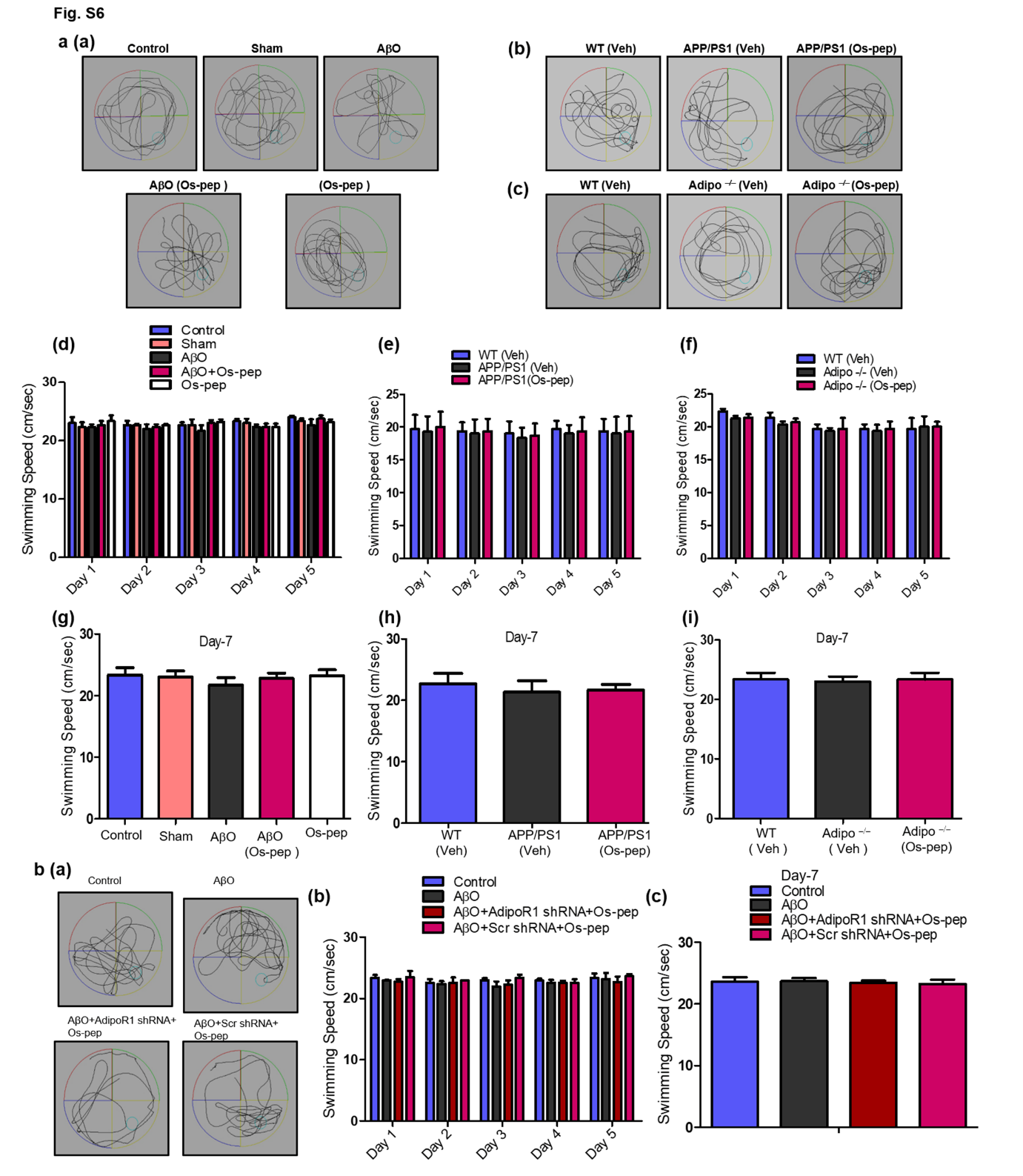
**

**Fig. S6. Path length and swimming speed of the AβO-treated, APP/PS1 and Adipo^−/−^ mouse models during the MWZ test. a, panle (a-c)** The path length of the mice during the probe test. **a, panels (d-i)** Histograms represent the swimming speed (cm/sec) of the mice during attempts to reach the hidden platform in the training session and on day 7 before the probe test. The swimming speed (cm/sec) was calculated with the equation v= distance/time. No significant difference in swimming speed was observed among the groups. **b.** **Path length and swimming speed of the AβO-treated mice (subjected to scramble and functional AdipoR1 shRNA) during the MWZ test.** **b panel (a)** The path length of the mice during the probe test. **b panels (b, c)** Histograms represent the swimming speed (cm/sec) of the mice during attempts to reach the hidden platform in the training session and on day 7 before the probe test. The swimming speed (cm/sec) was calculated with the equation v= distance/time. No significant difference in swimming speed was observed among the groups.
